# Part II. Hospital Reports

**Published:** 1827-04

**Authors:** 


*Foreign and Domestic;*

WITH COMMENTARIES.

### PART II.
### HOSPITAL REPORTS.

"*Ore trahit quodcunque potest, atque addit acervo.*"

### 1. PLEURISY AND ABSCESSES AFTER SURGICAL OPERATIONS.*
[Clinique de Perfectionnement.]

PART I

Every practitioner must have observed that, when a suppuration is established in a part, and another local inflammation set up in the same individual, a large suppuration is often established in the secondarily inflamed part with surprising rapidity. This is still more remarkable when the intercurrent phlogosis occurs in a part not tending naturally to suppurate, or where the product of inflammation differs from that of common suppuration, as, for example, in the arachnoid, the pericardium, the synovial membranes, the tunica vaginalis testis; and, above all, in the peritoneum and pleura. This species of superinduced phlegmasia is particularly deserving of the practitioner's attention, because it is one that frequently occurs; one whose causes, symptoms, and march are but little in consonance with those which we observe in other inflammations of the same organs or parts; and, finally, because the treatment must be different. It is in public hospitals, particularly after great surgical operations, and suppurations of any extent, that we have the best opportunities of witnessing facts of this kind. It is with the laudable view of inviting attention to, and throwing some light upon this important subject, that M. Velpeau has presented his brethren with some clinical reports in the present paper.

*Case* 1. Petrel, 39 years of age, of good constitution, and never before having experienced any severe illness, was received into the Hopital de Perfectionnement, on the 8th January, 1826. He had

* M. Alf. Velpeau. Revue Medicale, Decembre, 1826.

had his right thumb crushed the evening before. Abscesses formed deep in the hand, wrist, and fore-arm ; and ultimately such disorganization of parts took place, that it was necessary to amputate the arm on the 8th February. From this time till the 15th every thing appeared to do well ; but at the latter date, he had a shiver, and fever followed. The features became shrunk, the respiration was impeded ; yet the patient could lie on either side, had no cough, and felt no pain in any part of the chest. On the 16th he complained of sharp pain in the stump, extending to the shoulder. Suppuration was less free in the wound, and the parts looked unhealthy. The breathing still continued short, the pulse was small and irregular, but not very quick, the tongue moist and not coated. During the 17th and 18th the same symptoms continued, the debility increasing. On the 19th the patient died.

*Dissection.* The stump was flabby and unhealthy. The encephalon and its envelopes were sound. The abdominal viscera, examined with the greatest care, presented no trace of disease. The left thoracic cavity, however, contained a large quantity of thin creamy and puriform fluid, resembling a mixture of whey and matter, but containing no flocculent or albuminous materials, thus differing from those collections which we find after death in common inflammation of the pleura. On draining out the fluid from the cavity, the pleura was every where found covered with a coat of purulent matter exactly resembling that which had come from the stump, and without any trace of that albuminous concretion which we see in pleurisy. The membrane, being washed and cleaned, was scarcely altered from its natural appearance and polish. The lung of this side was reduced to one-third its natural dimensions. It was not inflamed. In the left side of the chest, every thing was sound.

*Case* 2. A young man, 26 years of age, had the middle finger of his right hand so badly crushed by a log of wood, on the 18th of May, 1826, that amputation was necessary some days afterwards. On the 6th, 7th, and 8th of June, the hand became swelled and very painful, with corresponding sympathetic fever. On the 9th there was an abundant suppuration. 10th. The inflammation and swelling reached the middle of the forearm. 12th. The matter was thin and of bad quality, but there was now very little fever—the abdomen was drawn in and tense—tongue white and moist. 13th and 14th. The disease made rapid progress, and two incisions were made, when a large quantity of pus escaped. The most attentive examination did not detect any disease in the thorax or abdomen. Nevertheless the patient was evidently hastening to the grave. On the 16th, in the evening, a considerable space of pleuritic inflammation was discovered by the stethoscope in the right side of the chest, and the respiration became very short. The pulse was neither fuller nor quicker than natural. A blister to the seat of inflammation. 17th. No pain was now felt in the right side of the chest—the breathing was free—but the chest sounded dull in that side, and no respiratory murmur could be heard there. 18th. The patient reported himself better, and complained only of weakness in consequence of starvation. For two days past the suppuration in the hand and arm has greatly diminished, and the soft parts of the member are flaccid, like those of a dead subject. The pulse is now extremely small. He died in the evening of this day.

*Dissection.* The hand and fore-arm were entirely disorganized, the skin, tendons, and muscles appearing as if macerated in dark pus. There was very little blood in the vascular system, and it did not appear natural. The cranial and abdominal organs appeared perfectly healthy. In the right thoracic cavity there was an effusion to an immense extent of sero-purulent fluid, like that in the preceding case, and exactly of the same nature as what came from the arm previous to death. There were at least five pints of this effusion in the bag of the pleura. This membrane throughout exhibited a layer of "veritable pus." When this last was removed, the pleura appeared red and inflamed in some parts, but quite natural in others. In the lung of this side there were found six tubercles, crude or suppurated, generally the size of a nut each, but some of them much larger, not surrounded by any inflammation. There was no hepatisation, nor was the mucous membrane of the bronchia inflamed. In the left lung there were two or three tubercles, but every thing else was sound.

*Case* 3. Rayer, aged 45 years, short of stature, and apparently of strong constitution, was admitted into the hospital on the 25th July, 1826. Excepting hæmorrhoids, this man had not been subject to any other disease. The neighbourhood of the anus and the lower part of the rectum presented tubercles that bled when irritated, and caused him great distress. These had continued for nearly a year. On the 30th July an operation was performed for the removal of these tumours. In the evening of the same day there was a profuse hæmorrhage which caused syncope. The bleeding ceased spontaneously, and was followed by a profuse perspiration. On the second day, the pulse had acquired strength, and there was no pain about the anus. On the third day there was fever, which continued during the fourth, but unaccompanied by any local pain. The dressings were removed, but nothing particular appeared in the wound, except some clots of half-putrid blood mixed with some suppuration, which came away from the rectum. On the 5th the pulse was improved, but the prostration of strength had increased. Some blood came away from the rectum. There was no thirst—the patient considered himself getting better—and could lie equally well on either side. On the 6th the debility increased; but there was no pain, nor any local affection complained of. He died in the evening of the 7th, the bowels having been always free—the breathing easy—and no cough till the last.

*Dissection.* The encephalon and its coverings in a state of integrity. The gastro-intestinal mucous membrane unaltered. Some of the lymphatic glands in the groin, and the cellular membrane in the neighbourhood of the bladder were in a state of suppuration. In the lungs and liver, upwards of thirty small abscesses, of various sizes, (which will be alluded to in the second part of this memoir,) were found. There were no traces of inflammation in the immediate vicinity of any of these abscesses, in either organ. In the left thoracic cavity, there was a considerable effusion of thin pus, not resembling the flocculent serous effusions which we see after unequivocal pleuritis.

*Remarks.* It would be easy to accumulate numerous cases of the kind here described; but it is unnecessary, as every man of observation must have been surprised at such unexpected occurrences. This pleuritic inflammation is peculiarly remarkable, in being unattended with the usual symptoms characteristic of the disease; and also in being speedily mortal, without corresponding phenomena indicative of the danger.

In respect to the explication of the pathology of these cases, we are perfectly agreed with M. Velpeau, that the fluids circulating in the vessels play an important part in the drama. In our own minds we have not a doubt that deleterious fluids are taken up by the absorbents; in cases where parts do not heal kindly after wounds and operations; and that these poisons give rise to the phenomena of metastasis in the vital organs afterwards. It is in this way, we think, that dissection and other poisoned wounds produce such havock in the constitution. The ill-conditioned pus which is generated in the parts, ultimately taints the whole system, and destroys life. In fact, we believe that, in these and in many other cases, as we have often observed before, the seat of disease is originally in the fluids.

### SECOND PART OF THE MEMOIR.

In the second part of his memoir, M. Velpeau treats of tubercular abscesses in subjects who have died after great surgical operations, and also after the suppression of a more or less abundant suppuration. Our author published a thesis on this subject, some years ago, and subsequent experience has only served to confirm him in the frequency of the disease. In the wards under Messrs. Boyer and Roux, it was ascertained that, of fifteen people who had died after great surgical operations, pus, either fluid or concrete, was found in masses of various sizes, and forming species of tubercle, in different organs of the body, in the following proportions:—viz. in ten, these purulent depôts were in the lungs—in two they were found in the liver alone—in three they were found in both the liver and lungs—and in one, in the spleen.

M. Velpeau thinks that the formation of these distinct purulent depôts, varying in size, from a hempseed to that of a pullet's egg, is characteristic of the resorption of pus from wounds or suppurations. They are formed with surprising rapidity, and yet without attendant symptoms which give notice of their existence. In the liver, these depôts are of greater magnitude than in the lungs, and they contain a greater variety of fluids or concretes. Although the signs of this dangerous translation to an internal organ are very obscure, our author thinks that such an event may be suspected when, in an individual, who has undergone a surgical operation, or who has been affected with ulcers, abscesses, or suppuration of any kind, there arises a sudden and unexpected reaction, without manifest or adequate cause. In such cases, we have generally rigors, which return at irregular intervals—the skin being dry, or hot, or covered occasionally with perspiration. The pulse is little to be depended on; but is usually frequent. There is generally cephalalgia, and the features are strikingly altered. In some subjects there is diarrhœa; in others, obstinate constipation—in others still, no affection of the abdominal functions. The external wound or suppuration, in the mean time, undergoes considerable changes. The soft parts collapse, and become pale and flaccid. The discharge becomes thinner, serous, and fetid. The quantity is often notably diminished. Hæmorrhages, under such circumstances, often appear unexpectedly; debility makes rapid progress; and death takes place from the fifth to the twentieth day. In cases of this kind, we may confidently predict that purulent pleurisy or purulent depôts have taken place.

In respect to treatment, our author thinks that bleeding is hurtful in the majority of cases; and it is not difficult to account for this, when we reflect on the original cause of the malady, and on the well-known fact that venesection promotes absorption. "Purgation would probably be more advantageous, as favouring a determination of fluids to the intestinal canal." The melancholy fact is, that death almost invariably takes place under such circumstances; and therefore the great object is to prevent the constitutional disturbance by diligent attention to the external wound or suppuration, and to the removal of the original source of the complaint—especially the stagnant fluids about the wound.

*Case* 4. Guillemin, aged 52 years, an old soldier, of apparently robust constitution, was admitted into the Hospital of the Faculty, on the 3d of May, 1826, having been long affected with disease of the ancle-joint. He came for the purpose of amputation of the limb. The operation was performed on the 7th of the same month, and union by the first intention was attempted. On the 8th the stump was painful, and sympathetic fever came on, but not severely. Low diet—lemonade for drink. 9th. The fever more developed, and in the evening an abundant perspiration broke out. 10th. Same state. The stump is hot and painful. Eight ounces of blood from the arm. 11th. The pulse is reduced, but the tongue is getting dry, and the thirst is urgent. Perspirations occasionally break out, and there are shooting pains in the stump. 12th. The dressings were removed—there was no union effected. Erysipelas appeared in the neighbourhood of the wound, and had extended to the knee. Dry lint and simple dressings were applied. The patient expressed himself as better. 13th. Same symptoms as on the 11th. The suppuration is abundant and very thin. The erysipelas appeared inclined to bound itself to the neighbourhood of the knee. 15th. The face was tinted yellow—the features shrunk—pulse small and unequal. The patient affirmed that he felt no uneasiness in any part but the stump, which was still inflamed. The abdomen was pressed in all directions, and the chest explored by auscultation, without detecting any disease. 16th. Some delirium, and somnolency. The suppuration in the stump is greatly diminished, and the parts are flaccid and hanging about. 17th. Comatose. 18th. He died.

*Dissection.* Several purulent collections were found between the stump and the hip, in the cellular substance connecting the muscles. There was much pus found in the veins of the limb. There was much suppuration in the cellular membrane about the hip-joint, and in the pelvis. The veins, up as far as the cava, where it enters the heart, contained pus mixed with blood. In the liver, four purulent depôts, the size of small eggs, were found. The pus was very thin, and exactly resembled the discharge from the stump. In some, however, the matter was thick, especially towards the centres of the depôts. The parenchymatous structure of the liver was unaltered. The mucous membrane of the stomach and bowels was sound, and the fæces in the rectum were solid and healthy. The thoracic and cranial contents were perfectly sound.

*Case* 5. Louvet, aged 27 years, strong and well made, having never before experienced any severe disease, entered the Hospital of the Faculty, on the 2d October, for a wound in the great toe of the left foot. The toe was divided, except a small strip of flesh. The parts were brought together, and union attempted. No inconvenience was felt during the first four days. The dressings were removed, and no union had taken place. During the fifth and sixth days there was pain in the toe, and some suppuration. On the 7th, inflammation was found to spread along the foot, with much pain. There was fever, and the wound looked unhealthy. The discharge is abundant and discoloured. Cataplasms, venesection. 8th. The fever is diminished. 11th. Heat and dryness of the skin—epigastrium morbidly sensible to pressure. 12th. In the evening, the patient complained of pain in the right hypochondrium—breathing difficult—abdomen rather tense—the conjunctivæ yellow—tongue and mouth dry. Twenty-five leeches to the region of the liver. 13th. Seemed better; but the abdomen is more distended. Twenty-five leeches to the epigastrium. Delirium in the night. 14th. The tongue is very dry in the centre, red and moist at the sides. The breathing is still more embarrassed. Skin very dry and hot. The abdominal pain augmented till the 18th. He died on the 19th, having had 40 leeches on the abdomen that day.

*Dissection,* The surface was jaundiced. The parts in the neighbourhood of the wound were much disorganized, and some purulent depôts in the foot. In the abdomen, the peritoneum was inflamed in several places, and some sero-purulent matter effused into the cavity of the abdomen. The alimentary canal was scrupulously examined, and found perfectly sound. A considerable portion of the convex surface of the liver was acutely inflamed, and covered with a crust of coagulable lymph. In the substance of this organ there were found several whitish masses, and several tubercles, not yet softened down. "The former were evidently collections of concrete pus, formed in the hepatic parenchyma." One of these whitish masses or depôts was as large as a pullet's egg, and projected against the diaphragm, which was, at that point, in a state of suppuration.* The portion of right lung opposed to the liver was inflamed, and even suppurated, and the neighbouring portions hepatized, with clusters of tubercles interspersed. There was also some inflammation and tuberculation in the left lung. The other viscera of the body were sound.

In M. Velpeau's remarks on this case, he adverts to the difficulty of accounting for these circumscribed purulent depôts in different organs, by simple inflammation of the parts. Indeed, we think it impossible to reconcile these depositions, in globular masses, with common phlogosis. There is some other kind of process, besides inflammation, to produce these and many other kinds of deposition in cavities and parenchymatous structures.

*Case* 5. Perrin, aged 38 years, of robust constitution, had contracted syphilis at the age of 26 years, and was properly cured. In 1823, being in his 38th year, he had his foot injured by a tight shoe, which injury came into the state of an ill-conditioned sore, for which he was brought into the Hospice, on the 23d September of the above year. The leg was inflamed and painful, and a bad-looking ulcer on the outer side of the instep. Bleeding by leeches and other means were used; but the ulcer spread, and gangrene commenced. The gangrene ascended, and ultimately reached as high as the knee, when amputation was performed; but whether a line of separation had, or had not taken place, is not stated. We rather think, however, that no line of demarcation had commenced. Things were not mended by the operation, and he died five days after the amputation, without exhibiting any sign of disease in the thorax or abdomen.

*Dissection.* The face of the stump was gangrened, and no adhesion had taken place. Trains of abscesses were found in the cellular membrane up along the thigh, and in some of the lymphatic glands of the groin. There was some inflammation, to no great extent, in the pleura. In the abdomen, there were several patches of inflammation on the peritoneum, and some sero-purulent effusion into the general cavity. Excepting some redness in one or two points of the mucous membrane of the stomach, there was no appreciable lesion in any part of the digestive canal. In the liver a number of abscesses were found distributed here and there through its substance. In these, the purulent matter presented various aspects, from a thin serous character to thick pus. The intermediate parenchymatous structure was not inflamed or otherwise altered.

* We can scarcely agree with M. Velpeau, that the white masses abovementioned were specifically different from the more concrete, or the ripe tubercles found in the same viscus.

*Case* 6. A young woman, 19 years of age, was admitted into the Hospital of the Faculty to be confined of her first child. This took place on the 1st February. The first four days passed without accident. On the 5th she had a shiver, followed by fever. On the 7th, the tongue was red at the tip and pale in other parts. The abdomen soft. The 8th and 9th in the same state. On the 10th the fever became stronger, and there was a slight cough, occasioning a very little pain in the thorax. This continued during the next two days. On the 13th, the fever is severe—face flushed—epigastrium sensible to pressure—cough troublesome—no expectoration. 14th. The breathing is sensibly embarrassed—fixed pain in the right side. Twenty-five leeches to the chest. 15th. The breathing shorter—the pain of side more diffused, but less acute—diarrhœa—a violent shiver.—The fever increased—the pain extended over a great part of the thorax, and the patient died on the 19th.

*Dissection.* The lungs were slightly hepatized, near the diaphragm, but no trace of pleurisy. In the abdomen, the peritoneum covering the liver was slightly inflamed. In this last organ there were some twenty purulent depôts, of the same kind as in the preceding case. The intermediate parenchymatous structure of the liver was not affected. The uterus contained a quantity of blackish matters, pulpy, or almost fluid. The veins of the uterus were found to contain the same material, and even the hypogastric veins. There was pus in the parietes of the uterus.

Our authors anticipate an objection which may be made, in this case, to their doctrine of resorption—namely, that there was no external wound from which pus might be taken into the circulation. They justly observe, that the internal surface of the uterus might very well serve the place of an external wound, and afford matter for absorption. Indeed, this last doctrine is maintained, as our readers know, by Professor Hamilton, of Edinburgh, as we have shown in our last number, page 223, *et seq.* The following is the resumé which our author has given.

"1mo. That those who die of acute diseases succeeding surgical operations, or profuse suppurations, generally fall victims to pleurisy and to the formation of abscesses, more or less numerous, in the viscera.

"2ndo. That this kind of pleurisy, hitherto undescribed, is of a peculiar nature, and might be denominated the "pleurisy succeeding operations."

"3tio. That this disease differs from simple pleurisy, in the latency of its march, the rapidity of its progress, and the almost invariable certainty of its fatality.

"4to. That the purulent depôts above described never acquire a large volume; but are circumscribed and spherical collections of matter, concrete or fluid.

"5to. That they are more especially developed in the liver and the lungs, where they may give rise to a species of tuberculous affection.

"6to. That the pleurisy and formation of depôts are rarely accompanied by characteristic local symptoms, sufficient to give notice of their existence.

"7timo. That these said inflammations and depôts are manifested under the influence of absorption of pus, and of its transport into the current of the circulation.

"8vo. That general bloodletting appears to favour the development of the maladies in question.

"9to. That, finally, where the existence of the malady was unequivocal, the march of the disease has hitherto been unchecked by any medical treatment employed."*

We recommend an attentive consideration of this paper to our professional brethren.

---

### 2. INJURIES OF THE HEAD.

[Middlesex.]

In the Medical and Physical Journal for January, is a report of some cases of injuries of the head, treated at the Middlesex Hospital. The first two are cases of scalp-wounds, and were under the care of Mr. Shaw.

*Case* 1. D. Maynard, æt. 63, was thrown from the box of a hackney coach, Nov. 7th, 1825, and received a severe scalp wound, extending from just above the right ear to the external angular process of the frontal bone on the same side, and then turning upwards and backwards to end at the vertex. The integument was much lacerated, and the bone extensively denuded of pericranium. The lips of the wound were brought together by adhesive straps and a roller, and calomel and jalap were given, so as to open the bowels freely. On the 10th, erysipelas appeared, but, by the 16th, it had declined: the wound was suppurating kindly, though its edges had receded, leaving a portion of bone, nine or ten inches in circumference, uncovered. All seemed to go on favourably till Dec. 5th, when the patient became irritable; the surface of the wound was dry, and a sanious discharge oozed from it; tongue furred. *Calomel and antimony.* 6th. He became delirious, and died in the night.

*Dissection.* Pericranium thickened, the bone beneath perforated by numerous holes, giving it a worm-eaten appearance. A small portion of bone, far removed from the centre of the external caries, was diseased throughout its whole thickness. The dura mater, at this part, had separated from the bone, and was thickened; the surface of the brain immediately beneath was *not* discoloured. Ventricles enlarged and filled with serum.

*Remarks.* A question arises: was this a case for the application of

* We think this conclusion is rather too hasty. The great fatality of constitutional disorder, resulting from resorption of ill-conditioned discharges and suppurations, is unquestionable; but we sometimes see recoveries from the worst forms of the disease. Whether these recoveries be spontaneous, or effected by art, we do not presume to decide.

the trephine? We apprehend not; for, setting aside the extent of the injured and diseased bone, we question much whether the death was not owing rather to the *general* injury done the brain in the erysipelatous attack, than the *local* mischief arising from the carious bone.

*Case* 2. — Henderson, æt. 37, was admitted, March 22, 1826, having the anterior part of the right parietal, with a portion of the frontal bones, denuded of scalp and periosteum; the surface dry and ash-coloured, with here and there a spot of caries. A fissure, large enough to admit the bulb of a probe, marked the recession of the living from the dead bone. The right eminentia frontalis was similarly diseased, and the whole was surrounded by dark-coloured granulations.

It appeared that the man was subject to epileptic fits, and that in one of these, about ten weeks previously, he had fallen with his head on the bars of the grate, occasioning a burn of the scalp which was followed by sloughing and exposure of the bone. He had now for the first time an attack of maniacal delirium, and subsequently he has had three more such attacks, usually lasting a couple of days.

His general health on his admission was much affected. He could not walk without assistance, and his intellects had suffered. On the 29th, he had his usual maniacal attack, "the symptoms generally resembling those of a patient labouring under acute typhus." On the 31st, he was becoming rational, the muscles of the face of the affected side being partially paralysed. April 1st, he became quite paralytic; the surface of the body was covered with petechiæ, and on the night of the 2nd he died comatose.

*Dissection.* A part of the dura mater, a little larger than a shilling, immediately beneath the larger portion of diseased bone, was separated and covered with a minute quantity of sero-purulent fluid; lymph had been effused on the surface of the membrane at this part, and to its inner secreting surface a small shred of lymph was found attached. No signs of inflammation in the arachnoid or brain, save that there was about an ounce of serum in the ventricles.

*Remarks.* Did the maniacal attacks in this case depend upon the carious bone? The reporter of the case thinks not, and we are inclined to agree with him, for he observes that the first paroxysm appeared before, in all probability, the bone could have been dead throughout, and Dr. Prichard observed that in inveterate epileptics, attacks of violent maniacal delirium not uncommonly supervene. At the same time it is not unlikely that the injury done the bone proved an exciting cause to the delirious paroxysms. We question too, although there was matter on the dura mater, whether the trephine could have been of any service in such an unfavourable case.

Three cases of concussion are related, one of them fatal. In this case the surface of the brain was found, on dissection, to be covered with spots of extravasation; and its structure apparently broken down at points, it being here and there softer than natural, with spots of extravasated blood; the cerebral vessels were turgid and dilated.

### 3. CLINIQUE OF THE HOTEL DIEU.

[M. Recamier.]

M. Martinet has reported the autumnal quarter of M. Recamier's practice in the above establishment, through the medium of the Revue Medicale for December, 1826.

The number of patients received was 144, of which 90 were discharged cured; 33 relieved or remained *in statu quo*; and 14 died—the mortality being thus one in ten. Of the whole number, 97 were acute, and 47 chronic diseases. We shall now select some of the observations contained in this report.

1. *Intermittents.* Eight cases of this fever occurred—all being contracted in Paris. Five were quickly cured by emetic tartar in ptisans, so as to act upwards and downwards. A disordered state of the digestive organs induced M. Recamier to prescribe this remedy. They were quotidian intermittents of recent origin and unconnected with any internal phlegmasia. The cephalalgia was the most troublesome symptom. In one of these patients there had been some pain in the abdomen, and the physician who attended him, in private practice, had him bled; but the accessions continued undiminished. A neighbour then prescribed a bottle of wine with plenty of pepper in it, which the patient took; but still the paroxysms continued precisely the same as after the bleeding. The man then repaired to the hospital, where M. Recamier put him on diluents and vegetable juices. In eight days the quotidian disappeared without any medicine.

One case of tertian fever was accompanied by tumefaction of the spleen, and gastro-biliary derangement. Two grains of tartar emetic were given in a pint of veal broth, with the effect of cleansing the primæ viæ and reducing the general malaise. Twenty leeches were applied to the left hypochondrium. The next paroxysm, which was the tenth, was much less violent than the preceding accessions. Twenty more leeches were applied, and the consequence was that the eleventh paroxysm lasted little more than an hour. The cure was completed in a few days by vegetable juices, eau de vichy, and tepid baths.

The second case of tertian, was at the 9th period of accession. The right hypochondrium was painful and somewhat tumid. Fifteen leeches to this part enfeebled the succeeding fit. Twelve grains of sulphate of quinine, given in divided doses during the apyrexia, prevented the return of the ague.

M. Recamier is of opinion that intermittent fevers are not always to be stopped with safety, at an early period, by the quinine or bark. We think he is right. We believe the disease, in this country at least, is much more frequently connected with, or dependent on, some local irritation, than is generally imagined. We know a young gentleman in this metropolis, who always has a paroxysm of ague when any fæcal matters accumulate about the caput or ascending portion of colon. Nothing will stop the paroxysms till these irritating matters are dislodged, when the ague disappears, without any bark or arsenic. Several other instances of a similar kind have lately fallen under our notice. The practitioner should, therefore, upon such occasions, make a strict examination into the state of the abdominal viscera, before he *throws in* the bark.

2. *Arachnitis.* Two cases only of this complaint are detailed or commented on. The first case was that of a man who had continued for more than a month, in a state of stupor and insensibility, with delirium and some febrile movements. As the symptoms were not violent, and had continued some time, M. Recamier contented himself with cold applications to the head. The sensorium, however, did not become free till a copious eruption of boils took place about the hips. The man then soon got well.

The second case was that of a man who had been seized with inflammation of the brain after a long exposure of the head to a powerful sun. He died. On dissection, the cerebral pulp was found to be altered in texture, especially in the neighbourhood of the septum lucidum, where the parts were softened. The arachnoid was infiltrated and thickened, as was the pia mater all over the superior portions of the hemispheres. Delirium was the characteristic feature of the man's disease during life.

3. *Paralysis.* Practitioners are aware that the paralysis which succeeds apoplexy, is generally owing to an extravasation of blood in the brain, and requires a long time for resorption. But then we occasionally see cases where the paralysis suddenly disappears, under different kinds of treatment, and in such a manner as to induce suspicion that cerebral extravasation could not have been the cause. In this uncertainty respecting the true state of the case, in paralysis, M. Recamier thought it might not be improper to employ galvanism in the case of a man, aged 42 years, who had laboured for two months under hemiplegia, the result of apoplexy. The motility and sensibility of both upper and lower extremities of one side were annihilated. The galvanic fluid was thrown from the spinal column in the neck through the arm, and also the leg affected with palsy. When the galvanism had been used about fifteen times, the motility of the lower extremity returned in some degree; but that of the upper was not at all bettered. Not so with the sensibility. This was restored completely in both extremities, by the use of galvanism. This fact is somewhat favourable to the remedy; for during the two preceding months no change whatever had taken place, in respect to either motility or sensibility, in the paralysed members.

4. *Prussic Acid.* This medicine was employed in about a dozen of cases of chronic catarrh or advanced stages of phthisis. The doses were, at first, two, three, or four drops in pectoral ptisans, in the course of the 24 hours. The quantity never exceeded six drops in that space of time. In three of the cases, the medicine produced no effect whatever. In the majority of instances, however, it induced a sense of heat and constriction in the throat and stomach—in a few instances, colicky pains. In one case of phthisis, it gave origin to severe diarrhœa, and was discontinued. In about half the subjects, the cough was notably diminished, as well as the dyspnœa and the difficulty of expectorating. The nature of the sputa, however, was not changed. The nights were less distressing under its influence, and some sleep was obtained. In one young man, the cough and fever were removed for a time; but a relapse took place, and death ensued. In these cases, other anodynes had entirely failed. Upon the whole, it appears evident to M. Recamier, and indeed to others, that the prussic acid, especially in large doses, irritates the mucous membrane of the digestive tube, and therefore should not be administered when this membrane is in a state of chronic inflammation or irritation. In other respects, it seems to exercise a considerable sedative influence on the respiratory apparatus.

5. *Hæmoptysis.* Several cases of recent hæmoptysis were treated with nitre, either alone, or combined with conserve of roses. The dose was, from a drachm to half an ounce in the 24 hours. It produced no inconvenience, in this quantity, in the stomach or bowels. The patients merely complained of an acrid heat in the throat, and the urine was sensibly increased. The hæmoptysis gradually diminished, and soon ceased altogether. M. Recamier observes that the practice of giving large doses of nitre, imported from Italy into France, is now established beyond all doubt, as a very useful one, in the disease above-mentioned, and in some others.

6. *Angina Pectoris.* A supposed case of this disease is detailed by M. Recamier, of which we shall state some of the particulars.

*Case.* Ravier, aged 37 years, had been subject, in early youth, to fainting fits and falls, from sudden loss of power in the lower extremities. About the age of fifteen, he became affected with palpitations and sense of uneasiness in the region of the heart; but his health was otherwise undisturbed till the age of 29, when profuse hæmoptysis occurred. This was checked by bleeding, leeching, and other means. It returned the succeeding spring, but did not continue long. Four years passed without any discharge of blood from the lungs; but the pain in the præcordial region continued, bounded to a space of about six inches in extent, over the sixth rib on the left side. The year after, although Ravier was gaining in embonpoint, both his arms, but especially the left, became the seat of a painful numbness, that stretched down, occasionally, to the fingers' ends, in the direction of the median nerves. To this was added a difficulty of breathing—all these symptoms coming on periodically. In 1824, the attacks were in the habit of recurring at short intervals, and it was found necessary to have recourse to repeated bleedings to mitigate the dyspnœa, which was now rendered very distressing on the least motion, especially of ascent. In process of time, the sufferer was unable to go to bed at all, and was afraid of going to sleep in any position. In this condition he came under the notice of M. Recamier, who made an accurate examination of the case, of which the following is a summary. The pulse is regular at both wrists, being full and not frequent. In the remissions (which were much prolonged in the hospital, by repeated bleedings) exploration of the chest by auscultation, percussion, &c. shewed nothing particular. The heart beats over a moderate space, and no unnatural noise is heard in that region. The respiration is free, and percussion elicits a natural sound in all parts. Nothing can be felt in the tract of the nerves of the arm, where the pain is sometimes so distressing. When the accession comes on, (which is not now very frequent in consequence of low diet and bleeding,) the pulsations of the heart become very violent, quick, and extended over a great space—the respiration is accelerated—the face reddens—and the præcordial and brachial pains become extremely intense, especially that of the left arm—the epigastrium is then so tender that the slightest touch cannot be borne, without convulsive motion of the diaphragm and imminence of suffocation. An accession of the paroxysm has more than once been brought on by pressure of the epigastrium. An attentive examination of the abdominal viscera could not detect any disease there.

M. Recamier does not venture to pronounce this a decided case of angina pectoris; but considers that it bears a very close resemblance to that terrible malady. We may observe, that there are essential differences in the symptoms, which induce us to place the above disease to some other account than that of angina pectoris. These are, the redness of the face and the increased strength of the heart's action during the paroxysm. It is directly the reverse in real angina pectoris. The face turns of a deadly pale colour, and the pulsations of the heart are small, quick, and confined or intermitting. We have had such repeated opportunities of watching the phenomena, and examining the bodies after death, in this fatal disease, (three exquisitely marked cases of which we have published) that we have no hesitation in separating the case of M. Recamier from those of syncope anginosa. We are inclined to look upon the present case as an instance of nervous irritation—and especially of irritation of the epigastric centre. The exceeding tenderness at the pit of the stomach, and the circumstance of pressure there bringing on an attack, are, in our minds, proof positive that the origin of the malady is in or near the solar plexus—or in the stomach or duodenum, where the nerves of this system are so profusely scattered. The case is interesting, and we hope it will be long ere the scalpel unravels its true nature. Indeed, we much question whether the knife is capable of unveiling the mystery.

7. *Intestinal Ulceration.* Our readers will see, in another part of this Periscope, an interesting case of intestinal hæmorrhage, during convalescence from fever, and which proved, on dissection, to be occasioned by ulceration of the ileum, the product of the preceding fever. The following case mentioned in M. Recamier's report, is somewhat analogous.

"Among the subjects who fell a victim to fever this quarter, we cannot pass over in silence the case of a young man who, having twice recovered from severe attacks, had a third relapse, from which also he emerged, for ten days, into a state of convalescence, and then suddenly sunk. On dissection, a chain of ulcerations was found in the ileum and cæcum. What great care should be taken, during convalescence from fever, to guard the patients against too much food! These people attribute all their debility to want of nourishment, and the consequences of repletion are too often fatal!"

In the case of a female, who died after being a week in the hospital, presenting the common symptoms of fever, some pain in the abdomen, stupor, and diarrhœa, the stomach and small intestines were found perfectly healthy; but, from the valve of the colon to the rectum there was scarcely a spot which was free from ulceration! The mucous membrane was almost entirely destroyed, and even the muscular coat, in numerous cases, was perforated. These cases may prove useful beacons to the young practitioner, when pressed by nurses and friends to allow food in early convalescence from fever.

---

### 4. CELLULAR INFLAMMATION.

[Bartholomew's.]

In a clinical lecture delivered by Mr. Earle, at Bartholomew's hospital, and afterwards published in the January number of the Medical and Physical Journal, we find some interesting observations on the treatment of this severe disease, especially as relates to the practice of making deep incisions, first introduced by Mr. Copland Hutchison.

Mr. Earle objects to the term phlegmonous erysipelas, which is usually applied to diffuse inflammation of the cellular membrane. Erysipelas is essentially of the skin, whereas the disease under consideration "exerts its influence (a bad expression by the way) principally on the subcutaneous tissue and fascia." For some time past, cases of this kind have been occurring at St. Bartholomew's in most of which Mr. Hutchison's plan was adopted, with success. The disease, as surgeons well know, is an acute and rapidly diffused phlogosis of the cellular membrane, to which no bounds are set, and which, if not arrested quickly, terminates in suppuration and sloughing of the subcutaneous tissues and fasciæ, which relieves itself, if the patient survives, by a large and uncontrollable sloughing of the integuments. Mr. E. has known, in the space of 36 hours, the whole integuments of an upper and lower extremity involved in one extensive sloughing abscess. In his experience, it has generally followed punctured wounds or severe bruises upon the elbow, and lacerations of the integuments of the hands or fingers. It has also occurred after wounds of joints, or in the neighbourhood of joints, which have been attempted to be closed. In the majority of cases, it was accompanied by well-defined inflammation of the absorbents; but these soon became so involved in the general tumefaction of the limb, that they could not be traced. The constitution sympathises much—the secretions are deranged or suspended—the circulation hurried—the pulse hard and contracted, and, after a time, irregular. The nervous system is greatly disturbed—the countenance anxious—features contracted—want of sleep. When the cellular membrane has sloughed extensively, the greatest prostration of strength takes place—and bad irritative fever too often closes the scene.

The treatment which Mr. Earle has found most beneficial was, by free longitudinal incisions, if possible, before suppuration takes place, through the swollen inflamed integuments, down to the fascia or muscles. The vessels of the skin should be allowed to bleed freely, and even encouraged by warm fomentations. The limb should then be enveloped in a warm bread and water poultice. A large dose of calomel, antimony, and opium is then to be given; and, after some hours, the patient should be freely purged with salts and senna. If the incisions be sufficiently large and deep, the relief is very speedy, and it is seldom necessary to repeat them. In a few hours there will be a complete subsidence of the tension and pain—the nervous system will be tranquillized—and the secretions restored.

The objections which have been urged against this practice, on the score of the severity of the operation, are frivolous and vexatious; but they are such as will always weigh with timid practitioners, or with those who have not confidence in themselves, or the art of inspiring confidence in their patients.

Fourteen years ago the Editor of this Journal drew the attention of the profession to this mode of treatment, in a quarterly report of sick and wounded on board the flag-ship of the late Sir William Young, in the Scheldt, and published in the 7th vol. of the "New Medical and Physical Journal, p. 183—203, March, 1814." We shall give a short extract from this report, in corroboration of the practice.

*H. M. S. Impregnable, in the Scheldt, Feb.* 1814.

"The species of erysipelas, which I have observed most prevalent in the navy, comes nearest to that which Dr. Bateman treats of under the head of 'Erysipelas Œdematodes.' When it attacks the face or breast, it is often dangerous. 'Vomiting, rigors and delirium, followed by coma, take place about the height of the disorder, and often terminate fatally on the seventh or eighth day.' Bateman, p. 127. Several instances of this kind I witnessed on board the Saturn a few years ago in Basque Roads, which proved fatal. Where it attacked the lower extremities, the inflammation soon destroyed the *cellular membrane*, forming the connecting medium between the integuments (including the adipose substance) and the muscles; which membrane, (attended with a quantity of ill-conditioned pus) would occasionally slough away, and come out wherever any orifices were made, either by nature or art, leaving a whole tribe of integuments surrounding, but disconnected from, the subjacent muscles. When this happens, in the thigh for instance, the patient is too often exhausted by the discharge, the mischief spreading farther and farther up, till it even begins to separate the muscles of the abdomen from the superincumbent integuments, and thus puts an end to his miseries! To check this evil, in embryo, a medical gentleman of my acquaintance, (Mr. Hutchison, of Deal Hospital) first, I believe, made deep incisions through the skin, adipose and cellular membranes, quite down to the muscles, as early as possible, and before suppuration. The effect is, a discharge of the stagnant-looking blood, with a reduction of the tension, swelling, heat, pain, and, in short, of all the unfavourable symptoms. I have witnessed this in three or four instances; and in one case lately, where the erysipelas was spreading fast up the leg, half a dozen incisions completely arrested its progress in one day, and in a short time afterwards the man returned to his duty. I am not prepared to say how far this treatment could be adopted, where the affection appears on the face, head, or breast; my observations being confined to the disease, as it appears in the above-mentioned manner, on the extremities; but I cannot help recommending these practical facts to the serious attention of the profession, as I am well convinced, that many lives and much suffering may be saved by adopting the treatment sketched out."*

We believe the above report was published some time before Mr. Hutchison's paper appeared in the Medico-chirurgical Transactions; and the cases alluded to in the Saturn, in Basque Roads, happened in 1809 or 1810. There the disease assumed an epidemic form, and several men died with typhoid symptoms. The incisions alluded to were made, in these cases, with great benefit; but they were often too late, as the destruction of the cellular membrane goes on with inconceivable rapidity.

Seven cases, in illustration, are given by Mr. Earle, for the particulars of which we refer to the journal in which they are published.

---

* In corroboration of the term *œdematodes*, as applied by Dr. Johnson, to the disease in question, we may cite a case reported from St. Thomas's Hospital, in No. 176 of the Lancet. A sailor, 27 years of age, of sallow unhealthy aspect, was admitted under Mr. Travers, on the 12th December, 1826, with diffuse cellular inflammation of the right upper arm. He had received a contusion, while intoxicated, some days previously. There was now a dusky redness of the skin, from the shoulder to below the elbow, and spreading over the breast. The limb was immensely swelled. "At some parts it had a tense unyielding feel, whilst at other parts, there was slight *œdema* on pressure." There was fever and other constitutional symptoms. Leeches in large numbers and lotions were applied, and he had a grain of calomel and a quarter of a grain of tartar emetic every four hours. A poultice was next day ordered. These remedies were continued for some days; but, on the 18th December, we find incisions made, which gave great relief, although only a small quantity of matter was evacuated. From the wounds, large sloughs of cellular membrane were extracted, and ill conditioned pus flowed out. The patient was now much relieved—the quinine was given, with wine and nourishing diet, and the man recovered.

The above was a case where early incisions would have saved the patient much suffering, and greatly abridged, if they did not entirely prevent the extensive sloughing of the cellular membrane.

### 5. M. LISFRANC ON ULCERS, SIMPLE AND VARICOSE.
(LA PITIÉ.)

The distinguished surgeon of LA PITIÉ has lately reported, through the medium of his pupil, M. Klimatis, an interesting memoir on ulcers, more especially of the lower extremities. M. Lisfranc does not accord in sentiment with those who attribute the frequency of ulcers on the legs to weakness of the vital powers in those parts. He places the cause of this affection to the account of "inflammatory irritation," determined by remora of blood in the saphena vein, which vessel presents between the malleolus and head of the tibia, in most instances, no valvular apparatus. When any valves are found, they are never seen between the malleolus internus and the calf of the leg. "It is precisely in this last place that the solution of continuity in question most frequently manifests itself." The reason is, he thinks, the difficulty which the blood encounters, in this place, in its return against gravity, unassisted by valves. The stasis of the venous blood becomes an irritant, and induces an inflammatory condition, which causes and keeps up the ulceration. The success which attends ligature of the saphena vein, in ulcers of the leg, irremediable by other means, is, he observes, an incontrovertible proof of the truth of his theory. The means employed by the partisans of atony and debility, furnish auxiliary proofs—as the horizontal posture, quietude, compression.

These ulcers are much more frequently situated on the left than on the right leg. How is this to be accounted for? The partisans of atony said it was because the left leg is naturally weaker than the right. But, of a number of ambidexters, or rather of left-footed patients, and consequently where the balance of strength was on the left side, it was still found that ulcers were infinitely more frequent on the left leg than on the right. M. Lisfranc accounts for the circumstance by the pressure of the sigmoid flexure of the colon, (so often distended with indurated fæces,) on the external iliac vein of that side. It is for the same reason that sarcoceles, cirsoceles, and hydroceles are more frequent in the left than in the right testicle, or side of the scrotum, the pressure of the sigmoid flexure acting on the spermatic cord, and retarding the return of blood.

M. Lisfranc has some peculiar notions respecting the nature of these ulcerations of the legs. He considers them a kind of gangrenous affection, (une affection gangreneuse, *sui generis*). We humbly conceive that this is but a very slight improvement on John Hunter's term—"ulcerative inflammation." Be this as it may, we all know the difficulty of healing old-established ulcers on the lower extremities, and the various means that have been tried in this and other countries for expediting such process. M. Lisfranc alludes to the real or supposed danger of closing drains of this kind long established. Many of the best observers in medicine and surgery have testified to the diseases which have supervened on such cures, especially to cerebral and pulmonary congestions; and consequently have recommended the opening of issues or other drains to prevent these accidents. M. Lisfranc believes that the danger arises from a bad condition of some of the viscera at the time the ulcer is healed, rather than from any disease which could result from the simple incarnation and cicatrization of a discharging sore. With this view, he accurately examines his patients, and when he finds any visceral disorder, he first endeavours to put that out of the way, by the proper remedies and by proper diet. He next corrects the bad state of the ulcer itself, by reducing inflammation and irritation, through the medium of rest, leeches, poultices, and other proper applications. When the sore is thus brought to a condition of being healed, he applies bundles of charpie wetted with solution of chloruret of lime or of soda, and keeps the sore soaked with such solution, graduated in strength to the sensibility or irritability of the parts. By this application he avers, and appeals to the wards of La Pitié for proofs, that he heals these old ulcers in a very short time, varying from five to eighteen or twenty days.

But when the ulcer is based on a lardaceous or disorganized substratum, this will not succeed. M. Lisfranc then tries ablations, by slices, of the disorganized tissue; or, more frequently he makes various incisions, crucial and perpendicular, into the parts. If these do not succeed, ablation of the whole is effected, by the knife or by the cautery, according to the extent and depth of the morbid structure. The actual cautery, in some obstinate cases, is substituted by M. Lisfranc, for the potential. When the ulcer is thus brought to a tenable condition, the chloruret is applied, and cicatrization soon follows.

Those who frequent La Pitié are surprised at the astonishing rapidity with which this able surgeon heals burns and scalds in this way. Forty-eight hours are often sufficient for healing of burns of the first or second degree of intensity. Ulcers healed in this way are said to be infinitely less liable to break out again, than those treated in the common manner. He properly advises the laced stocking or other support, however, after the cure thus performed. And, in cases where there is any tendency to visceral or cerebral disorder, he establishes one or more issues on the upper or lower extremities.

The chlorurets will not be successful where the ulcer is connected with, or dependent on a varicose state of the veins of the limb. The ligature and section of the saphena vein have been often practised in this country and in France; but dreadful accidents have too generally ensued. The sides of the vessel are usually so thickened that they will not collapse when cut; and if air gets in, a rapid and destructive phlebitis is established. M. Lisfranc therefore abandoned this practice, and cut out two or more inches of the dilated vessel, trusting to careful compression of the vein above and below the resection, rather than to the ligature. In this manner he has operated a great number of times in La Pitié, and no fatal accident has ever succeeded such practice. As success, in this operation, depends on the formation of a coagulum in the saphena, it is necessary to select a spot for the operation, where there are no collateral communications. M. Lisfranc generally selects the inside of the knee, near the upper extremity of the tibia, for the resection, the branches of communication being all below this joint. If the saphena be varicose up as high as the knee, this state almost always coincides with a chronic inflammation of the sides of the vessel. In such case, M. Lisfranc performs the resection on a sound portion of the vessel, on the inside of the thigh, but as low down as possible.

*Operation.* The patient is placed horizontally, and if it is the internal saphena that is to be operated on, the limb is laid, a little bent, on the external side. An assistant compresses the vein, by grasping the limb tightly above the part. The surgeon then, with a bistoury, makes an incision along the vessel, going only through the skin. When the vein is laid bare and insulated, the surgeon introduces the point of the scissors under the vessel, close to each angle of the wound, and snips the vein across. A considerable quantity of blood is then suffered to escape, if the vessel will bleed, in order to prevent inflammation ; and if hæmorrhage will not take place, the patient is bled from the arm. Light, but careful pressure is then made above and below the wound, which is healed, if possible, by the first intention. Much pressure ought not to be made on the wound itself, for fear of irritation and inflammation. The patient should be kept quiet, and the utmost attention paid to diet and regimen. In less than thirty hours, the ulcer changes its aspect, from red or violet to pale—the purulent secretion diminishes, and assumes a healthy appearance. Cicatrization sometimes follows, in five, ten, or fifteen days, but the medium term is from twenty-five to thirty.

Phlebitis may occasionally succeed the operation. The saphena will become tense and painful, feeling like a cord under the finger. If not checked, the inflammation will run upwards, and death may be the consequence. M. Lisfranc, in such cases, abandons the inflammation of the saphena below the wound to itself, and covers the track of the vessel above the resection with leeches. When these fall off, a warm poultice with plenty of laudanum is applied. Since he trusted entirely to bleeding and poulticing *above* the wound, he has never lost a single patient. We shall close this paper with a case in illustration.

*Case.* Tellier, aged 40 years, entered La Pitié on the 10th of November, having on the inside of the left leg a varicose ulcer, the size of a man's hand. This ulcer had continued for several years, and was accompanied by a multiplicity of varicose veins, and very callous edges. It had resisted all kinds of treatment, and therefore the operation was performed on the tenth of the same month, in the manner already described, an inch and a half of the vessel being removed. The upper extremity of the vein furnished a considerable quantity of blood, and slight compression was employed. 16th, The ulcer is pale—there is no pain—and the patient appears in a good state. 17th, The upper portion of the vein is painful and hard. Twenty-five leeches were applied, followed by cataplasms, with laudanum. 18th, No amelioration of the phlebitis. Twenty-five leeches again applied. 20th, Some traces of the phlebitis still remained, and another batch of twentyfive leeches were put on the track of the vessel, the cataplasms and anodynes being continued. 21st. The inflammation has completely disappeared. The poultices were still persevered with. The ulcer is red, the suppuration good, cicatrisation is commencing. 25th, The patient was so well that food was given him. He was discharged cured on the 27th January.—*Revue Med. Decembre*, 1826.

As a proof of the great success which attends M. Lisfranc's treatment of ulcers by means of the chloruret of lime, and resection of varicose veins, it is stated that La Pitié usually contained about 50 of these cases constantly within its wards; whereas there are now very few seen there, so speedily are they cured and discharged. We think our hospital surgeons may take some useful hints from their Parisian confrere; and that they may profit by this paper, and an article on the chlorurets in a preceding part of the present number of this Journal.

### 6. INJURIES OF THE HEAD.

[Middlesex.]

In the February No. of the Medical and Physical Journal, the subject of injuries of the head is continued, and some interesting cases are brought forward, shewing the difficulty of ascertaining the precise condition of the brain and its membranes in these accidents. We shall notice some of these cases.

*Case* 1. A boy, 11 years of age, had fallen from a considerable height, and was taken up senseless. He was bled, and then sent to the hospital, 6th Dec. 1826. He was still insensible, after an hour had elapsed. He was cold, pale, and pulseless, with dilated pupils, and tranquil respiration. There was a denuded portion of cranium on the vertex, the size of a shilling. In the course of another hour, he shewed returning sense, and cried loudly when his head was touched; but when unmolested he sunk into a state of stupor. His right leg was found to be fractured. The pulse had become perceptible, and animal heat was returning. Head shaved, and eveporating lotions applied. Calomel and jalap administered. 7th, Much the same. Moans, and is very irritable. More purgatives, as the medicines had not acted. In the evening, the bowels were freely opened. Saline medicines with antimony. 8th, Has been noisy in the night—talks incoherently—pulse still weak, and only 76. Leeches to the temples—calomel and antimony at bed-time. 9th, Is constantly crying—passes his urine and fæces involuntarily. Pulse sharp, though weak. Leeches to the temples—a dose of calomel and jalap. 10th, Face flushed—skin hot and dry—tongue foul—pulse thrilling, 85 and stronger. Venesection to 4 ounces—and 12 leeches to the temples. 12th, Still continues noisy and sleepless—pulse softer after the last bleeding, but is again vibrating. V. S. ℥vj. From this time he gradually improved.

*Case* 2. A boy, aged 10 years, was admitted, 24th Aug. 1826, with all the usual symptoms of compression, caused by the fall of a heavy piece of timber on his head. He was pale, cold, motionless—pulse small and feeble—constant vomiting. Scalp contused over the middle of the parietal bone, without wound—no irregularity in the bone to be detected by pressure. Head shaved—cold lotions—purgatives. 25th, Bowels have been well opened—lies comatose—skin has become hot and dry—face flushed—pulse quick and strong—*breathing perfectly easy*—irregular action of the irides—can be roused for a moment by loud speaking—thighs and legs drawn up—asks for drink, or the pot de chambre—rolls about his head and moans—often raises his hand to his head. Venesection to ten ounces, which partially restored his senses; but the favourable change was only temporary. The vomiting still continues. Sulphate of magnesia in small doses. 26th, Became convulsed at 4 o'clock to day, and died.

*Dissection.* The parietal bone was found to be fractured, and a small portion slightly depressed. From this part a fissure could be traced backwards along the parietal bone, and terminating near the base of the skull. About two ounces of dark coagulated blood were found on the dura mater, under the fractured and depressed portion of the skull. Two branches of the meningeal artery had been torn, but the dura mater was not perforated. The part of the hemisphere under the coagulum was flattened, and softer than the surrounding parts. "There was a dark spot on the surface of the brain, which corresponded to the depressed portion of bone." There were no marks of inflammation in the brain.

It is remarked here that experience has failed, hitherto, to point out the distinction between concussion and compression. It may be so. But, when such symptoms as the above take place, with an obvious contusion of the scalp, are we not authorised to cut down and examine the state of the bone at least; and even if no external fracture can be detected, are we not authorised to trephine, under such circumstances as the above? Can it be asserted that the removal of a piece of bone, and also the ablation of the coagulum pressing on the brain, would not have saved this boy's life? Surely such pressure was adequate to the production of the symptoms detailed, and may be fairly considered as the cause of death.*

* In the same number of the Medical and Physical Journal, there is a case related by Mr. Boyle, Surgeon of the Middlesex Infirmary, which bears upon this point. A boy, nine years of age, was thrown from a horse, on the pavement, and was supposed to have fractured his skull. On examination, a large open space was seen, commencing at the situation of the anterior fontanel, and widening as it ascended on the right side, in the course of the sagittal suture. This was stated to have existed since birth. "The symptoms were, a total suspension of the mental powers, stertorous breathing, dilatation of the pupils, with insensibility to light, a low, rapid and irregular pulse, coldness of the extremities." Marks of external violence were detected behind the posterior superior angle of the right parietal bone. On pressure being made here, a depression was felt. The integu-

*Case* 3. P. C. a strong Irish labourer, fell, July 12th, 1826, from a scaffolding thirty-five feet in height. On admission he was quite insensible; pulse scarcely to be felt; pupils much dilated; respiration unembarrassed. He rolled about in bed, often groaning and putting his hands to his head, and lay with the thighs drawn up towards the abdomen and head bent upon the breast. Scalp apparently uninjured—considerable hæmorrhage from the left ear. *Head to be shaved—calomel and jalap.* The pulse continued weak and not above forty all day, and he remained in a dosing state. 13th, Much the same, save that there is some heat of scalp. *Hirud.* xij. *temporibus—cold lotion to the scalp—purgative enema, as the calomel has not operated.* In the evening the pulse got up to fifty, and was stronger; the bowels had been opened, and though he was roused once, he relapsed into a lethargic state. *Emplast. canthar. nuchæ.* 14th, Rather more sensible; pupils more contracted, but he was still restless. *Calomel and jalap—aloetic enema.* In the evening the pulse got up, and he was bled to eight ounces with relief. 15th, Sensorial functions very imperfectly performed; pain in the head; pulse quicker; bowels not opened. *Aloetic glyster to be repeated, with house medicine—ten leeches behind the ears—calomel and antimony three times a day.* From this time he continued slowly to improve under local bleeding, blistering, purges and antimonial medicines. *Aug. 5th,* He could walk in the garden with assistance. Aug. 15th, He was discharged. "He could then walk alone—he was quite deaf in the left ear; his intellects were much impaired; his countenance was a complete blank; in short he was little better than an idiot." Since then his faculties are in a slight degree restored.

*Remarks.* Was this a case of pure concussion? We think it was not. The bleeding from the left ear—the subsequent deafness in that ear—the extreme slowness of recovery, and the injury done the intellectual functions seem clearly to indicate compression on the brain. In fact we think it extremely probable that a vessel was ruptured, most likely the meningeal, that part of its contents escaped by the ear, and part coagulated on the surface of the brain producing the symptoms narrated above. Then comes the question of trephining; if the scalp be injured in any one place, there is some sort of guide to the surgeon,

---

ments were much tumefied. The pericranium was divided by a crucial incision, and the dura mater exposed, this being a part originally unprotected by bone. Effused blood was found pressing on this membrane, and removed. A considerable hæmorrhage occurred. The boy was now able to answer questions, and the pupils contracted on the application of light. Leeches, cold lotions, purgatives, and other very proper means were employed, and the boy recovered. When all swelling of the integuments had subsided, it was found that there was a very considerable portion of brain unprotected by bone. We think the treatment was very judicious, and that the removal of the extravasated blood from the dura mater, contributed mainly to the successful issue of the case.

but if, as here, the scalp be *uninjured*, and the bone unbroken, it might be dangerous practice to *dig* in search of coagula upon the dura mater.

Before closing this short article, we may also notice a case of compound fracture of the cranium [in the Lancet] from Guy's Hospital. The boy (12 years of age) had received a kick from a horse on the head, by which the bone (parietal) was comminuted, and the scalp irregularly wounded. The symptoms of compression were present, as stertor, slow pulse, dilated pupils, insensibility, &c. Under these circumstances, Mr. Bransby Cooper operated and removed the depressed portions of bone, by means of Hey's saw and the elevator. The patient very soon experienced relief. The pulse rose in a quarter of an hour, and consciousness returned. In the evening, there was reaction, and blood was abstracted. A brisk dose of calomel was given. The patient rapidly recovered. This was certainly the kind of case which promises most success under an operation, viz. fracture and depression of bone—laceration of integuments; in short, a compound fracture of the skull.

### 7. CONGENITAL DISLOCATION OF THE HIP-JOINT.*

[Hotel Dieu ]

It is well known that the most frequent kind of dislocation to which the hip-joint is liable is that of the head of the femur upwards and backwards on the dorsum of the ileum. It is also well known that of this dislocation there are two varieties—the first is the result of accident —the second is *consecutive*, the result of scrophulous ulceration in the joint. But M. Dupuytren in the memoir now before us, proposes to add to the list a third variety of this same dislocation, which, as it is found at birth, he terms "congenital."

The signs which characterise it, are shortening of the limb—presence of the head of the femur on the dorsum ilei—prominence (saillie) of the trochanter major (?)—retraction of almost all the muscles of the upper part of the thigh towards the crest of the ileum, where they form around the head of the femur a kind of cone, the base towards the os innominatum, the apex towards the trochanter—the almost entire denudation in consequence, of the tuber ischii—the rotation of the limb inwards—the obliquity of the thigh proportioned, of course, to the age and development of the pelvis—the meagreness of the limb, out of all proportion to the trunk and upper extremities, which are really well developed—and the imperfect motions, particularly of abduction and rotation. The upper part of the trunk of persons thus affected is thrown backwards whilst the lumbar portion of the column projects as much forwards; the pelvis is placed almost horizontally on the femurs, and the ball of the feet alone touches the ground. In walking we observe them incline the body strongly towards the limb which is to support the weight, at which moment the head of the femur of that side is seen distinctly to rise on the dorsum ilei, in consequence of the superincumbent weight and sinking of the pelvis, and then they drag painfully

* Baron Dupuytren; Répertoire, No. 3, Tome 2me, 1826.

forwards the opposite limb, the head of the femur of which is perceived not to rise but to sink in consequence of its own weight drawing it down. This series of phenomena then is repeated each step the patient takes, and although locomotion to him is not so painful as it appears, still he is incapable of making any thing like a long journey.

In the recumbent posture, most of the symptoms of the dislocation in a great measure disappear, in consequence, no doubt, of the relaxation of the muscles and removal of the weight of the trunk. In this position of the body the surgeon can by a slight effort elongate the limb and shorten it again, that is, he can pull the head of the femur downwards or press it again upwards to the extent of two or even three inches, according to circumstances.

Let us look to the history of this complaint. Even at birth the prominence of the haunches, the obliquity of the femurs, &c. are perceptible, but in these cases the attention of the parents is seldom much directed to the malformation till the child begins to walk, and indeed even then its aukward efforts are attributed in general to weakness, &c. till the end of the third or fourth year, when the parent is at last convinced there must be something wrong. As the pelvis begins to be developed (for it is a curious fact that the growth of the pelvis is never affected in these patients) the symptoms which we have enumerated above become more marked, especially in females, and a person not acquainted with the true nature of the malady would consider it the consequence of scrophulous disease of the joint. But the previous history, the absence of all pain, swelling, abscess, fistula or cicatrix, and the simultaneous affection of both sides are sufficient to correct this error. At the same time it must be remarked that these individuals are for the most part of a lymphatic and scrophulous habit.

As the age of the person increases, and the superincumbent weight becomes of course greater, the heads of the femurs rise on the dorsum ilei till at last they almost touch the crista, the obliquity of the bones is increased, and the difficulty of motion proceeds at last so far as to incapacitate the patient from all active exercise.

As this of course is not a fatal disease, the opportunities for cultivating its pathology must be comparatively rare. In the cases which he has examined M. Dupuytren has found the acetabulum almost entirely obliterated or even entirely wanting; the head of the femur a little flattened on its internal and anterior surface, and a sort of cotyloid cavity to lodge it, formed on the dorsum of the ileum, as happens in unreduced accidental dislocations. In one or two instances, he has seen the ligamentum teres elongated, and in some places worn apparently from the pressure and friction of the head of the femur.

The Baron puts some questions to himself and the reader as to the etiology. He asks, can the dislocation be consecutive to a disease which assailed the fœtus and disappeared before birth? Or can it be the result of an ante-natal accident? Or lastly, can it be owing to an original imperfection of the acetabulum, in other words, can it be a congenital malformation? He seems to incline to this position, but like a skilful disputant he has dealt his blows so equally to all his men of straw, that it is almost hard to say which is up or which is down.

M. Dupuytren makes some very sensible remarks on the treatment, which of course can be but palliative. As the weight of the trunk is the main agent in aggravating the displacement, repose is obviously indicated; but it is not necessary to confine patients to the recumbent posture; for in the act of sitting there is no stress on the femurs, the body resting entirely on the tuberosities of the ischia. Let these individuals then choose a profession which they can exercise when seated. Our author advises, likewise, the use of the cold bath, and the application of a bandage which encircles the pelvis, confines the trochanters, and keeps them of an uniform height, thus binding the ill-adapted parts together, and preventing that continual motion to which they are exposed. This practice, though it certainly will not cure the complaint, will give a great degree of support to the hip-joints, and prevent the progress of the displacement.

In the course of eighteen years, M. Dupuytren has met with twenty cases of this kind, seventeen or eighteen of which have been females.

There is a plate appended, which shows the characters of this congenital dislocation very satisfactorily.

---

### 8. DISSECTION WOUND.*

[Mr. Shaw—Middlesex.]

This was the case of a medical gentleman, who, while sewing up a subject that had died of peritonitis, grazed with the needle the middle finger of the left hand. He did not notice this scratch at the time, but awoke the next morning (7th January) with pain and stiffness of the fingers and hand, the former soon extending up to the axilla. To these symptoms were added head-ache—dull heavy coldness of the other hand and of the feet. Feeling nervous and depressed, he took two table-spoonfuls of brandy in a cup of coffee, and then went out to visit some patients. The pain increased, and he had alternately a kind of hysterical fit of laughing and crying. Returning home his breathing became oppressed, with a sense of constriction in his throat, and difficult deglutition. He took some more brandy, as the sense of coldness augmented. By mid-day he had taken three quarters of a pint of brandy. A medical friend advised him to discontinue the stimulant—to take some calomel—and to apply some leeches to the chest. Mr. Shaw visited the patient about one o'clock, and found the wrist and hand of a livid colour—a similar patch on the inside of the arm—the finger swollen—but the wound scarcely perceptible. "His countenance was very extraordinary, his expression being that of a mixture of hysterical alarm and intoxication." The patient said he was a dead man, and felt that nothing could save him. Mr. Shaw gave immediately eight grains of calomel and six of colocynth, with two grains of opium, wrapping his arm and hand in a lotion of laudanum and liq.

---

* Med. Journal, Feb. 1827.

plumb. acet. dilut. Laudanum was ordered to be kept poured over the cloth as it dried.

Being a little composed Mr. S. left him, and returned in an hour. The patient was still composed, and he ordered a linseed-meal poultice made with a very strong solution of opium, to be applied to the hand and wrist. In the evening, Mr. Griffiths and Mr. Shaw visited the patient. They found him low and dejected, but quite composed. The hand and arm were not so much inflamed as in the middle of the day. Ten grains of Dover's powder and four of calomel to be taken immediately, followed by a draught of camphor mixture, carbonate of ammonia, and laudanum. The draught to be repeated every four hours. On calling next morning, they were not a little surprised that the "dead man" had gone to a considerable distance, to attend a woman in labour! They returned at 3 o'clock, and found their patient sitting comfortably in his parlour. At seven in the evening, he was so well as to give Mr. Shaw a clear and connected account of all that had passed up to the time of Mr. S's first visit. He had not even a head-ache, after so much brandy, though unused to spirits—the hand and arm were shrivelled, white, and free from pain. His bowels had been freely opened. He was advised to take some Dover's powder and opium, and a glass of brandy in gruel for supper. Next day he was out seeing his patients, and had no farther inconvenience.

We agree with Mr. Shaw that, although this gentleman had a rapid recovery, he had also a narrow escape. Indeed there can be no rational doubt that a specific principle had been absorbed in this case, for it would be preposterous to attribute all the phenomena to "thecal inflammation." They were far more like those which follow the bite of venomous animals, and we think they were of the same nature.

The treatment must be modified by the symptoms. With the deadly coldness and nervous dejection presented in the above case, stimulation was evidently the proper remedy. But a general rule cannot be founded thus. Had this gentleman presented symptoms of violent reaction and inflammation, the stimulating plan would have been improper, and even moderate depletion might have been justifiable. Here, in short, as in all other cases, we must be guided by the symptoms. High excitement must be controlled—nervous depression counteracted, by cordials and stimuli.

### 9. ANEURISM FROM VENESECTION.*

[St. George's.]

Two cases are related from the practice of Mr. Brodie at St. George's, of aneurism of the brachial artery following venesection. The first case, that of J. Rogers, will be found already detailed, at page 194 of our last number. Of the second case we shall give some particulars.

H. Chambers was bled in the West Indies in 1820, soon after which a pulsating tumour appeared at the bend of the arm, which by degrees attained the size of a large walnut. In April, 1820, a surgeon tied the brachial artery above the tumour. This diminished in size, and the

* Med. and Phys. Journ. for February, 1827.

pulsation ceased; but there came on numbness of the hand, and severe pain, which, however, subsided in a few weeks. In the course of two or three months, the tumour returned, and in September, 1820, he entered St. George's.

On pressing the aneurismal tumour, there was felt, besides the common pulsation, a peculiar thrill like that given by a varicose aneurism. Dec. 7th, Mr. Brodie tied the brachial artery, an inch and a half above the aneurism, when the pulsation ceased, but, in a few minutes, returned. A tourniquet was placed on the arm, the sac cut into, some coagula removed, and its inner polished membrane displayed. On loosening the tourniquet, blood flowed through two orifices, that of the upper and that of the lower portion of the brachial artery; consequently it was tied immediately above and below the sac. But there was yet a third orifice, through which flowed blood, partly venous and partly arterial, marking the communication with both an artery and vein. Into this the blunt end of a probe was passed, so as to mark its situation, and a ligature was then applied close to the sac, thus cutting off the communication of the vessels with it. All went on well, and in a month after the operation, the patient quitted the hospital cured.

This, no doubt, was a case which does occasionally happen, of complication of varicose with common aneurism. We think it might admit of question, whether, in such a case as this, the application of a ligature, above and below the sac, without cutting into it, would not be sufficient. This operation would bring the case into the same circumstances as simple varicose aneurism, which we know does not, in general, require a ligature; and would certainly be attended with less hazard than the opening of the sac. At the same time, we freely confess that the operation performed by Mr. Brodie *must* be more effectual, and *did* prove quite unattended with danger, in the above instance.

---

### 10. SURGICAL REPORTS OF ROUX, BRESCHET, AND DUPUYTREN.*

[Hôpital de la Faculté.]

In No. 10 of this Journal, we noticed the practice of M. Roux, in several cases, as recorded by M. Velpeau. We shall now lay before our readers the sequel of the above reports, drawn up by the same gentleman.

*Case* 1. This was a boy, two years of age, whose urachus remained open, forming a livid, fungus-like tumour at the umbilicus, which by pressure could be returned into the abdomen, and which discharged a fluid exactly resembling urine. The urinary organs themselves were well formed. Various means were tried to stop the unnatural discharge but without effect, neither catheters in the bladder nor plugging of the umbilical opening proving successful.

---

* Hôpital de la Faculté. Archives Générales, August, 1826.

*Stone in the Bladder.* Six cases of this fearful disease are detailed; five of them fatal, which is certainly a high ratio of mortality.

*Case* 1. The patient was a child, æt. 6, of good constitution, who had suffered from stone for two years. M. Blandin operated after the manner of F. Côm, but it is stated in the report, that the incision was so small that the lithotome and forceps were with difficulty introduced. Peritonitis supervened, and the boy died within 48 hours after the operation.

Here the smallness of the incision and consequent violence were no doubt the main cause of the fatal event.

*Case* 2. A boy, æt. 16, who had laboured under the symptoms of calculus for eleven years. In consequence of the size of the stone, M. Roux decided on performing the operation of Celsus, as modified by Béclard and Dupuytren. A semi-lunar incision was made about an inch anterior to the anus, *(cornibus ad coxas spectantibus paululum,)* and the neck of the bladder divided on both sides by the lithotome of F. Côm. The calculus, however, was so large that it was necessary to break it into fragments, and the extraction was tedious, and accompanied with a great degree of violence. On the following evening fever and cephalalgia appeared; these symptoms by the next day were much relieved, but they returned, and on the fourth day from the operation the patient sunk.

*Dissection.* General but slight peritonitis; infiltration of sero-purulent matter into the cellular membrane of the pelvis; parietes of the bladder reddened.

Would not the high operation have been advisable here? We know that in youths the outlet of the pelvis is comparatively more confined than in the adult, and consequently the extraction of a large stone difficult, and proportionably dangerous. This being the case, we think the high operation preferable to the one put in practice here, as the suppuration in the cellular membrane seems to have been principally owing to the force necessarily employed in extracting the masses of calculus from the bladder.

The next case we shall notice is more remarkable for the candid manner in which it is related than for any great éclât which attaches to the operator himself.

*Case* 3. This was a child, æt. $2\frac{1}{2}$, who for a year had suffered from uneasiness in the penis, and pain on making water. On sounding, M. Roux thought he felt a stone, but on the operating table, on sounding again, the results were less satisfactory. M. Velpeau and others introduced the instrument, and could not satisfy themselves of the presence of a calculus. M. Roux, however, thought he felt one, and the recollection of a former case where he alone had discovered a small calculus in the bladder, determined him to operate. The operation was performed with Hawkins' gorgeret, but no stone could be found. Peritonitis succeeded, and in 60 hours the child was dead. No examination of the body was permitted.

When such candour is shewn as in the report of the present case, one feels reluctant to employ the pen of criticism. We must say, however, that placed as M. Roux was, himself not satisfied of the existence of a stone, and those around him still less so, the performance of the operation was imprudent, to say the least of it. He is a confident man who *sounds* with a *gorget*. M. Velpeau remarks, that such instances are not so rare as, from the little mention made of them, one would be inclined to believe. Candour, no doubt, would be beneficial to the interests of science; but we much doubt whether in England at any rate, where public opinion is so free, we might almost say censorious, men will be found to publish to the world what may be any thing but advantageous to the interests of themselves.

*Case* 4. This was a diminutive lad of 15, and by no means a good subject for the operation, as he had a most unconquerable dread of it. The gorget was used; the stone found to be very large, and after having been broken to pieces, extracted with difficulty. The lad died on the second day.

*Dissection.* Purulent infiltration of the cellular membrane of the pelvis—fragments of stone still left in the bladder, and indeed in the wound; the surface of which was coated with coagulable lymph.

In lithotomy, more perhaps than in any other surgical operation, as much depends upon the constitution of the patient, his habits of life, and other fortuitous circumstances, as upon the skill and dexterity of the surgeon. At the same time, there are very many instances where the fatality is attributable to obvious causes, and we conscientiously think that in several of the above cases such is the fact. In the first case particularly, the smallness of the incision, and consequent violence necessary, as we observed before, left few well-grounded hopes of success. Upon the whole, we do not think these favourable specimens of lithotomy.

As we are upon the subject of calculus, we shall amalgamate with this report of M. Roux's the report on the same subject published by Messrs. Breschet, Dupuytren, and Sanson, in the Répertoire. These Gentlemen determined on making experiments at the Hotel Dieu, on the comparative merits of the transverse or bilateral operation, performed by M. Dupuytren; the lateral operation of F. Côm, performed by M. Breschet; and the recto-vesical operation, by M. Sanson. The different methods were put in practice *alternately*, and consequently without selection of cases.

*Transverse Operation.* The instruments M. Dupuytren uses are—a common staff, a lithotome somewhat like that of F. Côm, but consisting of two curved blades, which open in opposite directions by means of two levers, *(bascules,)* which are pressed against the handle of the instrument. The handle is moveable upon a screw, and so contrived that the separation of the blades can be regulated at will to the extent of eighteen lines. The mode of operating will be shewn in the following case.

*Case* 1. Rouzet, æt. 38, of lymphatico-sanguineous temperament, who had suffered pain in making water for thirty-two years, entered the Hotel Dieu, on the 14th June, 1825, for incontinence of urine. On sounding, M. Dupuytren discovered a stone apparently large and rough. It appeared that he had been in the habit of introducing the fingers of his left hand into the anus, in order to relieve the pain which he experienced. The patient was prepared by baths, light purges, and regimen, for the operation, which took place on the 1st July.

The patient being placed in the usual position, a slightly curved transverse incision, the concavity of which looked backwards, was made through the skin and cellular membrane, with the point of a straight bistoury, about seven or eight lines from the anus. M. Dupuytren fearing that the great dilatation of the lower part of the rectum (caused by the repeated introduction of the fingers) would expose the gut to injury from the blades of the lithotome, introduced two fingers of the left hand into the intestine, stretching it transversely, and pressing it towards the coccyx. A second and deeper incision was now made down to the urethra; this was followed by a good deal of bleeding. The urethra was next divided in the direction of its axis by the bistoury passed on the nail of the fore-finger. The double lithotome was opened to No. 15, and divided from right to left, and from behind forwards, the neck of the bladder and internal parts of the prostate. This was followed by much struggling of the patient; the gorgeret and forceps were introduced; the stone broke and was extracted piece-meal; it was as large, when whole, as a hen's egg, and appeared to consist of uric acid. Hæmorrhage to the extent of eight ounces occurred; three injections were thrown up, and a canula and plug of linen around it introduced into the wound; the whole was then covered with a T bandage. It is needless to go into the details of this case; suffice it to say that all went on well. By the 7th suppuration was established in the wound; for some time the urine continued to flow through it, but at last it was voided wholly by the urethra, and on the 10th August the patient was discharged perfectly cured.

*Case* 2. Scache, æt. 46, of sanguineous temperament, entered the Hotel Dieu, Sept. 5th, for calculus, to which he had been subject, more or less, since the age of seven years. For some time before admission he had been in the habit of passing, at intervals, urethral calculi, and about a month before this period he had passed a lumbricus eleven inches and a half long, giving rise to the suspicion that there existed a communication between the bladder and rectum.

On sounding the bladder, M. Dupuytren discovered a stone, which, on the 14th, was removed in fragments by the operation, as described above, without any hæmorrhage. In the evening cephalalgia came on, which was removed by bleeding. Next day colicky pains—bleeding to eight ounces, emollient cataplasm. From this time he continued slowly to improve, under the use of pills composed of Venice turpentine, gr. xl. acetate of lead, gr. iv. extract of white henbane, gr. vj. At the latter end of October he was discharged cured; the urine, however, still being slightly catarrhal.

There is nothing particular in the operation of M. Breschet. Both cases did well.

*Recto-vesical Operation.*—*Case* 1. Varnet, æt. 19, of melancholic temperament, entered the Hotel Dieu on the 4th October, 1823, with symptoms of stone of three years' standing. On sounding, calculus was easily discovered. Oct. 10th. The operation was performed by M. Sanson in the following manner. The staff being passed into the urethra, the membranous portion of this canal was divided by a longitudinal incision, carried from behind forwards on the median line of the perineum. Into this incision, a probe-pointed bistoury was introduced, and the prostate, neck of the bladder and its lower wall divided to the extent of about fifteen lines above the anus. A large stone was then extracted. No accident whatever happened, except that, in spite of cauterization, the wound in the rectum remained open, and loss of power to retain the urine was the consequence. M. Sanson has learnt that, since his discharge, the wound is contracting, and in a fair way to cicatrization.

The next case did well—the wound healed perfectly, and the patient was discharged cured in a month from his admission.

*Remarks.* M. Royer Collard, the reporter, observes, on the subject of hæmorrhage, that when internal, it may be recognized by these symptoms: the patient feels weight and uneasiness in the region of the bladder, arising from its distension; frequent desire to make water, which, however, he cannot pass; the cheek becomes blanched, at times even fits of syncope succeed, from which the patient recovers only on the accession of fresh pain. On examining the hypogastrium, a tumour will be felt, formed by the distended bladder. To stop this hæmorrhage, plugging, as was employed in the first case, is had recourse to. A silver or platina canula, three inches long, and five lines in diameter, terminating at one end in a cul-de-sac, pierced by numerous holes, is to be procured. At some distance from this extremity, a covering (*chemise*) of fine linen is attached, the canula introduced through the wound into the bladder, and between it and the *chemise* lint is to be stuffed, sufficient by its pressure to stop the hæmorrhage. The canula is to be secured by tapes and a bandage. If the bladder be already full, it is necessary to clear it out by injection, before introducing the canula.

From the small number of cases here detailed, and from their being all fortunate ones, no very accurate inferences can, of course, be drawn, as to the comparative merits of the several operations. Sufficient, however, has been brought forward on former occasions, as well as on this, to satisfy us that the recto-vesical operation is decidedly objectionable. The occurrence of fistula after its employment is not only likely to happen, but in at least five cases out of ten, actually does happen. There are six or seven cases adduced here, to show that these fistulæ will often, after a certain time, take on the healing process. But, granting that the fistulous communication remain open only for a year or so, the case is still bad enough in all conscience; and there is a chance of the disease continuing for a much longer period. The recto-vesical operation will never come into general use.

We shall now return to the Clinical Report of M. Roux, at the Faculté.

Two cases of strangulated hernia are related, the first an inguinal, and successfully operated on: the second, crural, and fatal, in consequence of the laceration of the intestine by the upper pillar of the ring, in the endeavour to draw it out. On examination after death, the knuckle of intestine was found not gangrenous, but so soft as to be very easily torn through. This should teach a surgeon caution in handling a strangulated gut. The operator was Mr. Wessely, an English student.

*Tumours.* Larochette, æt. 27, blind and deaf, had a large steatomatous tumour removed from the region of the right parotid gland on the 16th April. All went on well—the wound was nearly cicatrized, when the patient grew weaker, and died, without any symptoms of internal disease. On dissection, all were surprised to see the marks of a most intense peritonitis.

*Case 2.* Durand, æt. 44, robust, but of a highly irritable constitution, entered the hospital on the 4th May, with an encysted tumour, the size of a turkey's egg, on the forepart of the right knee. This was removed by M. Velpeau on the 5th, under the direction of M. Roux. Tumefaction of the limb, fever, great excitement, irritability, and even delirium succeeded, and in spite of bleedings and anodynes the patient expired on the 10th.

*Dissection, 36 Hours after Death.* Putrefaction had made remarkably rapid progress. The veins of the limb operated on were inflamed to a very trifling extent, and the cellular tissue infiltrated with serum. The veins of the dura mater were rather congested, but no other morbid appearance was to be seen. On dissecting the tumour, it was found to be a bursa mucosa, its structure altered, and containing partly its proper synovia, and partly a fluid more pultaceous, and seemingly the product of inflammation.

*Remarks.* This case exemplifies the mischievous consequences which will occasionally, though rarely, attend operations on the bursæ mucosæ. The degree of irritation produced in patients of nervous habits by the removal of these bodies is sometimes astonishing, whilst in others they may be treated very roughly, without the slightest ill effects. The patient in this case evidently died of the high degree of constitutional excitement produced by the operation, for there is nothing in the post-mortem appearances to account for the fatal result.

*Bleeding Polypus.* Massenet, æt. 13, entered the hospital, Nov. 25th, with a polypus of eight months' standing in the left nostril. It had been tied once and extirpated twice, and each time a considerable hæmorrhage occurred. M. Roux decided on extirpating it, and seized it with the forceps, but such was the toughness of the tumour that the forceps broke. The pedicle was then cut across by a straight probe-pointed bistoury, when such a hæmorrhage occurred, that it was necessary to suspend the operation and plug the nostril. Fever appeared, sore throat succeeded, the patient became comatose, and died on the 6th day from the operation.

*Dissection.* The tumour was attached to the back part of the roof of the nose—it was fibrous—of a reddish-gray colour—little vascular, but so tough as not to be torn through with the fingers. The bony palate was necrosed, and its mucous membrane, with the gum, detached; pharynx inflamed; pus between it and the spine, and the upper vertebral articulations themselves much altered.

Seven operations for cataract took place, only four of them successful. M. Velpeau apologises for this, by saying that the majority of the subjects were in a condition by no means favourable for extraction, the method which M. Roux generally adopts.

*Inflammation of the Muscular Coat of the Bladder.—Case.* Maurican, æt. 44, was suddenly seized, on the 5th February, with violent pains in the hypogastrium and difficulty of making water. He was admitted on the 8th. Nausea, small pulse, hypogastric pain, but no swelling. Twelve ounces of limpid water were drawn off by the catheter; the symptoms, however, increased, and on the 10th he died.

*Necropsy.* The bladder was found contracted, thickened in its parietes to the extent of an inch, and in the midst of its muscular fibres purulent matter was effused. There was also infiltration of pus in the cellular membrane around, and the peritoneum in the pelvis was inflamed.

Some cases are here detailed, where injuries about the hand were followed by excessive inflammation of the synovial membrane and sheaths of the tendons, ending in sloughing, death of the bones, and a fatal issue, in spite of amputation of the limb. These cases it would be needless to particularize, especially as M. Velpeau promises a memoir on the subject.

We have now concluded our analysis of this paper, and have little to add to what we have remarked on some of the individual cases. We may observe, however, that this report is drawn up in a very ingenuous and candid manner, that the practice is detailed as it really was, and that even where errors or oversights have been committed, they are stated fairly and manfully. This is as it should be.

P.S. In the second number of the Répertoire, appears the continuation of the series of experiments on lithotomy, instituted by M.M. Breschet, Dupuytren, and Sanson, at the Hôtel Dieu. During the three months which have elapsed since the last report, M. Breschet and M. Dupuytren have each performed their respective operations twice, and with success. There is nothing particular in these cases, and it would be useless to detail them here. The fifth case, however, which occurred to M. Sanson, and in which he performed the recto-vesical operation, we think worthy of notice.

*Case.* Duplessis, æt. 65, entered the Hôtel Dieu, March 31st, 1826, with strong symptoms of stone. He was decrepid beyond his years, his respiration was hurried, his voice feeble, the least muscular effort occasioned dyspnœa, and the pulse was feeble and irregular. The suffering on making water was excessive. On sounding, a calculus was distinctly felt in the bladder. On the 8th or 10th of April, the lithontriptic process was attempted by M. Meirien, in the presence of M.M. Breschet, Dupuytren, Sanson, and the éléves of the hospital; but, after five and twenty minutes spent in fruitless endeavours to seize the stone, the attempt was given over. Some fever, with incontinence of urine, ensued, and these symptoms were followed, on the 13th, by a swelling of the left testicle. Leeches, lotions, &c. were applied, and by the 1st of May the organ had regained its natural size. On the 13th of this month the recto-vesical operation was peformed by M. Sanson in the manner we have described above. The stone was extracted with little difficulty; its long diameter was about 16 lines, its short about 10, and its surface was very rough. Some degree of depression of the system took place after the operation, but on the next day a slight re-action followed, which, however, was checked before night. Pain in the hypogastrium, irregularity and intermissions of the pulse, cephalalgia, and torpidity of the bowels now shewed themselves, the urine flowing almost entirely by the wound. From the 20th to the 25th the patient was better, complaining of little else than the irritation of the skin produced by the dribbling of urine from the wound. Obstinate torpidity of the bowels again appeared, the skin around the anus became excoriated, and, finally, ulcerated; extreme prostration of body and mind supervened; solid fæces collected in the rectum and were obliged to be eked out by a spatula—the tongue became dry—the abdomen exquisitely tender, and on the 13th June the unfortunate patient expired.

*Dissection.* The peritoneal cavity contained between two and three pints of an opaque, flocculent fluid—the convolutions of the intestines were agglutinated by inflammation, and the large intestine was distended with solid fæces. The bladder itself was natural, except that its mucous membrane was a little coloured. The lips of the wound in the urethra, prostate and bladder were wide apart, in fact no attempt at adhesion had taken place. The prostate itself was so altered by suppuration that it was impossible to distinguish the ejaculatory ducts. All the cellular membrane of the pelvis was one vast abscess. The right kidney was soft and much altered, the left nearly as much so. Cavities of the heart dilated, and their walls ; aorta at its origin covered with numerous patches of ossification; about an ounce of serum in the cavity of each pleura.

*Remarks.* It would be hardly fair to cite this as a case which proves against the recto-vesical operation, for we much question whether any operation would have succeeded here. A man of 65, with symptoms of disease of the heart or large vessels, and a highly irritable habit, is no very good subject for lithotomy in whatever way performed. But still, the more we see, or rather read of M. Sanson's method, the more we are convinced that it neither will, nor deserves to, become general. The recoveries are uniformly tedious, fistula not an uncommon consequence, and this case has shewn, that it may produce extensive suppuration in the cellular membrane of the pelvis.

### II. CANCER OF THE PENIS.*

[Clinique de La Pitié.]

M. Lisfranc was often surprised, on amputating the penis, to find that the disease had not extended nearly so far as it appeared to do previously to the operation; and consequently he was led to the idea of making, in future, a kind of exploratory operation, before he ventured to remove the organ in toto. This exploration consists in making, on the dorsal face of the penis, an incision, parallel with the *axis* of this organ, commencing at the anterior part of the carcinomatous portion, and carried backwards to its posterior. The knife should be directed slowly along the parts, cutting by light strokes through the degenerated mass. By carefully sponging, the surgeon will thus come down (as if cutting on a hernial sac) to the fibrous envelope of the corpus cavernosum penis. If this be found sound, a careful dissection of the diseased parts may be effected, and the necessity of amputation avoided. If, on the contrary, the disease is ascertained to have spread its roots too deeply, the organ must be sacrificed. M. Lisfranc thinks that the addition of pain which this exploration occasions should not be put in competition with the chance thus afforded of preserving the organ. We shall now proceed to the statement of two cases in illustration.

*Case* 1. John Roussel, 27 years of age, was admitted into La Pitié, on the 6th of June, 1826. Some months previously he had had a paraphymosis succeeding a gonorrhœa, and which required an operation to set it free. The part had continued painful ever since, notwithstanding the use of antiphlogistic and antisyphilitic medicines. The pain afterwards became lancinating and insupportable. When received into hospital, the prepuce was observed to be retracted behind the gland, forming a dense, red ring, the thickness of a person's thumb,

* Sur le Diagnostic des Divers Degrés de Profundeur des Cancers de la Verge, &c. Par M. E. Margot. [La Pitié.] *Revue Medicale, Decembre,* 1826.

and apparently making part with the body of the penis. There were two points in a state of ulceration, discharging a thin fetid pus. The pain was very distressing, the pulse hard and quick. Twelve ounces of blood were taken from the arm—emollient poultices were applied—and the patient put upon low diet. These means were continued till the 10th, when an operation was practised. Every thing was prepared for amputation of the penis, in case the malady was found to amalgamate with the corpus cavernosum. A longitudinal incision was made, in the manner before-mentioned, from the posterior to the anterior portion of the tumour, penetrating slowly down to the fibrous envelop of the corpus cavernosum, carefully sponging away the blood as they proceeded. It was clearly ascertained that the said fibrous envelop was sound. It was therefore determined to dissect away the whole of the disease, which was no easy operation. The patient was intractable, and it was necessary to be careful in removing the whole of the disorganized mass, without injuring the body of the penis. Opposite to the two ulcerated points it was found that the fibrous envelop was partially diseased. The diseased portion was removed, and the whole mass finally dissected away, without injuring the urethra. Some vessels were next secured, and the operation completed. Simple dressings were applied, and the patient carried to his bed. Considerable inflammation and sympathetic fever followed, and were met by blood-letting, antiphlogistics, and poultices. On the 13th, suppuration commenced, and the symptoms were mitigated. Every thing went on favourably; but as it was feared that there was a syphilitic taint in the constitution, the proper remedies to correct this were administered. In order to effect a good cicatrization, the dressings were carefully attended to, and the chloruret of soda was used, in weak solution. By the end of July, the wound was completely healed. The patient has since been seen, and was found perfectly well.

*Case* 2. J. Chevalier, aged 46 years, entered La Pitié, on the 25th July, 1826, for an old-standing cancer of the penis, occupying the whole of the anterior part of the scrotum, the skin covering the pubes, and half of the penis. The right testicle was indurated and enlarged, and the spermatic cord in the same state. The disease had continued long; but the man was so deaf, that little information could be obtained as to the exact history of the case. The patient experienced almost constant lancinating pains, and ulceration prevailed far and wide over the diseased parts. The ulcers had raised and everted edges, with foul yellow sloughs at bottom. The pulse was hard and quick; but the appetite had not failed. He was bled from the arm—his diet reduced—and diluent drinks ordered. The ulcers were poulticed. These means were continued till the 5th of August, when the following operation was performed.

A semi-elliptic incision was made, beginning at the crural arch of the right side, an inch from the spine of the os pubis, and ending at the lower part of the scrotum; then carried upwards to the summit of the left testicle. Another incision, commencing from the same origin as the former, descended, and swept round to meet the other incision, in a manner which cannot well be described in words. M. Lisfranc then dissected away the diseased parts, with great care not to wound the spermatic cord or the testicle. Considerable hæmorrhage occurred during the operation, which was exceedingly difficult. In order to remove the cancerous disease of the penis and integuments over the pubes, incisions and dissections were carefully and adroitly made by M. Lisfranc. On the penis itself, a longitudinal incision was made through the diseased parts, to ascertain how far the roots of the cancer spread. They were found, in some places, to penetrate the fibrous envelop of the corpus cavernosum, and these points were removed either by excision or scraping with the edge of the scalpel. The operation was necessarily tedious and excessively difficult; but was, at length, brought to a conclusion, and dressings applied. Strong fever rose after this terrible operation; and next day it was thought proper to remove the dressings. The wound was considered to be in a good condition; "mais cette enorme denudation était affreuse à la vue." The patient was bled, and kept very low. On the 7th the fever was abated; but pain was great, and the wound was red and tumid. Forty leeches were applied to the abdomen and around the wound. Emollient poultices were applied. The penis was greatly swelled; but the swelling was œdematous. By the 11th suppuration was established, and the wound beginning to clean. Some nourishment was allowed. On the 14th the dressings were wetted with the solution of chloruret of soda. By the middle of September the whole surface was healed, and M. Lisfranc presented the patient to the Royal Academy of Medicine, the members of which verified the completion of the cure.

We think M. Lisfranc deserves a civic crown for thus preserving the life of a fellow-creature, who, but for this most arduous operation, was doomed to a lingering and torturous death.

---

### 12. RHEUMATIC OPHTHALMIA.*

[Glasgow Eye Infirmary.]

In a former number of our contemporary, Mr. Mackenzie offered some observations on *catarrhal* ophthalmia, (No. 11, Med. Chir. Rev. p. 173,) and now takes up the subject of *rheumatic* inflammation of the eye.

1. In respect to this last, it is seated in the albuginea, sclerotica, and periosteum, within and round the orbit.

2. *Redness.* This is zonular, and seated under the conjunctiva, whereas, in the catarrhal ophthalmia, the redness is reticular, and the turgid vessels are evidently conjunctival.

3. In the catarrhal ophthalmia the mucous membrane is inflamed, and there is an increased secretion of mucus. The rheumatic species attacks the fibrous membranes of the eye, and is unattended by any morbid secretion.

4. The *pain* of the catarrhal ophthalmia is compared to the feeling

* Professor Mackenzie. Med. and Phys. Journ. Jan. 1827.

of sand or roughness between the eye-lids—does not extend to the head—and is felt more in the morning, or when the eyes are moved: —the pain of the rheumatic species is pulsative and deep-seated—the chief pain not being in the eye, but round the orbit, in the eye-brow, temple, cheek, and side of the nose. It is severely aggravated between sunset and morning.

Compared with the catarrhal, this species is rare—perhaps one to ten. Its exciting causes may generally be traced to currents of cold air during perspiration, or to cold, in some way applied. It is more seen in middle, than in early or late periods of life. In general, there is no tendency to chemosis in the pure rheumatic ophthalmia, nor do the eye-lids take part in the disease. It is uniformly attended with dimness of vision, slight contraction of the pupil, and sluggishness of the iris. One eye only is, in general, affected, especially at first. The iris becomes slightly discoloured, and the attending iritis may go on to effusion of coagulable lymph within the pupil. The iritis in these cases, however, is not generally very severe. Mr. M. has not seen the disease terminate in any form of suppuration or of ulceration. The access of light is not very distressing. The eye is at first dry and hot; but after blood-letting and proper means have been used, there is a considerable epiphora. The pain is of a stinging kind, extending to the whole of the orbit, and greatly increased by heat. Occasionally it is confined exactly to one half of the head. It never ceases entirely while the disease continues; but it varies much in degree, coming on with severity about six o'clock in the evening, and being at its acmé about midnight.

Symptomatic fever accompanies the pain, rising *pari passu* with it. The digestive organs are deranged, the bowels confined, and the excretions morbid.

*Treatment.* Differing from Mr. Wardrop, our author insists upon general and local bleeding. Mr. W. has found little benefit from leeching—Mr. Mackenzie has found great advantage in this measure. Surgeons will differ. Perhaps their patients are of different constitutions. The banks of the Thames and the banks of the Clyde present different aspects. Those who reside on them are not less different in constitution. Mr. M. bleeds from the arm the first day, and the next applies leeches to the temple. If the vascular action continues strong, and the symptoms unrelieved, he repeats the venesection.

*Calomel and Opium.* This combination has never failed to prove highly useful, "in checking the circum orbital pain, restoring the digestive system to its healthy state, exciting the skin, and dissipating the redness of the eye." Two grains of calomel with one of opium are to be given every night till the gums are affected, when the calomel may be omitted, and ten grains of Dover's powder substituted.

*Opiate frictions* are serviceable—a large *blister* to the nape of the neck is of much benefit. Topical applications to the eye are of no use; but when the febrile and painful symptoms have abated, the vinum opii may be used. The pupil of the eye ought to be kept under the influence of belladonna all the time. Gentle aperients—diluents—and in the chronic stage, tonics, especially the arsenical solution, will be necessary.

Three well-marked cases are given in illustration, from the journals of the Glasgow Eye Infirmary. These we need not touch. We shall be happy to see Mr. Mackenzie's paper on the third species, or catarrho-rheumatic ophthalmia.

---

### 13. CHIRURGICAL CLINIQUE OF SAINT-LOUIS.

[M.M. Richerand and Cloquet.]

This report is drawn up by Mons. Hutin, (interne des hôpitaux) and commences with a very interesting case of intestinal suture, or entero-raphia, the particulars of which we shall now state.

1. *Operation for Hernia—Wounded Intestine.*—Nicholas le Jume, 41 years of age, entered the St. Louis Hospital, on the 13th of July, 1826, presenting the following symptoms to the house-surgeon:—inguinal hernia, on the left side, very large—pain in the abdomen—colick—constipation since the rupture became strangulated (early in the same day)—frequent vomiting—breathing laborious—pulse small, quick, and irregular—features shrunk. The taxis had been ineffectually employed before he came to the hospital, and afterwards by the house-surgeon. M. Cloquet having also tried to reduce the hernia, without success, proceeded to the operation. After laying bare and dividing the hernial sac, several convolutions of intestine presented themselves, greatly distended with gas, the intestinal coats being red and thickened. He proceeded to enlarge the opening of the ring, by the bistoury, carried on the point of the forefinger, upwards and outwards. In withdrawing the knife, an assistant let go a knuckle of intestine, which suddenly glanced on the edge of the instrument, and the consequence was, a transverse incision in the gut, full an inch in extent. Gas and fæcal matters rushed out in abundance. The operator instantly determined to take this opportunity of putting into execution the plan of M. Jobert, who had made a great number of experiments on animals, with the view of trying the results of ligatures applied to intestinal wounds. The protruded intestine was first emptied of its gazeous and other contents, and then three interrupted sutures were made, at convenient distances, across the wound. The plan adopted by M. Jobert, (who assisted at the present operation) is to plunge the point of the needle in at a little distance from the wound, and then bring it out again near the edge of the cut:—the same is done (inverting the order) at the opposite aide of the wound, and thus, when the knot is tied, the peritoneal surfaces come in contact, and a kind of seam is left on the internal surface of the gut, in the line of the wound. Adhesive inflammation soon glues the sides of the wound together, when thus stitched with the peritoneal surfaces in contact. The ligatures being thus applied, the ends were cut close off, and the hernia was returned into the abdomen. In a few hours the patient's bowels acted, the pulse rose, the countenance improved. In the evening he complained of pain in the left iliac region, and of tenderness in the abdomen generally. Twenty-five leeches were applied to the region above-mentioned, together with emollient fomentations, enemata, &c. The pain went off, and he passed a good night. 14th. Some return of abdominal pain this morning. Leeches were again applied, and the bowels were freely opened. A few days afterwards the bowels became confined, but without any bad symptom, and a dose of castor oil brought away abundant evacuations, From this time, the cure went on prosperously, and the patient recovered without a single accident. Since his discharge from the hospital, he has been seen several times, and continues in good health.

2. *Nitrate of Quicksilver.* This metallic preparation is coming into considerable notoriety in France, and especially in the St. Louis Hospital, as a caustic in scrofulous, syphilitic, and even cancerous ulcerations of the face—and in various cutaneous eruptions, which, says the reporter, " have yielded, as if by enchantment, to the action of this new remedy." Some cases are detailed of the efficacy of the nitrate, but we need not insert them here. The nitrate of mercury is a powerful escharotic, and the nitrico-oxyde of mercury has long been employed in this country as a remedial agent of great efficacy, in the form of the unguentum hydrarg. nitrico-oxyde.

3. *Acetate of Lead, as a Sedative in Cancer.* A number of females, affected with incurable and most painful cancers of the rectum or uterus, have had their sufferings greatly mitigated by warm baths, in each of which was dissolved an ounce of acetate of lead.

4. *Cranial Fractures.* We have hitherto been accustomed to regard the Continental practice, in regard to sanguineous depletion especially, as very inert. The French are now growing very bold on this point, and probably may soon rival their British neighbours.

In respect to fractures of the cranium, accidents which are very frequent in the St. Louis Hospital, the reporter observes that, the danger does not arise from the mere injury to the bone, but from the injury which the brain sustains then or afterwards. The following is the treatment pursued in the above institution. When the patient is received, he is bled largely—not to 20 or 24 ounces—but to the extent of 80 or 100 ounces—" vingt à vingt cinque palettes"—after which, derivatives and diluent drinks are prescribed.

*Case, in illustration.* Piron, a young man of strong constitution, a stone-mason, fell from a scaffold on his head, and was immediately conducted to the hospital, when the surgeon on duty, M. Paillard, found him presenting the following symptoms :—a large wound, with contused edges, on the posterior and lateral part of the left side of the head, denuding the lambdoidal suture, and part of the occipital bone, which was fractured in several directions, and the loose fragments, many of them, depressed. The breathing was slow and deep—pulse small and irregular—face pale—all parts of the body strongly convulsed—penis in a state of erection, and the intellect deranged. The patient was bled to twenty palettes (eighty ounces)—sinapisms were applied to the feet. The convulsions ceased—the erection of the penis disappeared. Next day the breathing was free—pulse calm—intellectual functions restored. On the fifth day, the patient was so well that he wished to leave the hospital, notwithstanding the fracture of the cranium. It was with difficulty he could be restrained from leaving the institution before the wound was well, which it was in a very short space of time.—*Bibliothèque Medicale, Novembre.*

The above was bold practice, considering the state of the pulse, and condition of the surface; but the end justified the means. It is intended to state a series of cases, exemplifying this decisive system of depletion.

---

### 14. CONSTITUTIONAL PREDISPOSITION TO SCIRRHUS.

[St. George's.]

Under this head, Mr. Jeffreys has published some cases, in the February Number of the Medical and Physical Journal. The predisposition in question is a melancholy pathological fact, which tends to check the sanguine hopes of the operator, when removing external tumours of the scirrhous character. We shall glance at some of these cases.

*Case* 1. A woman (aged 45) was admitted into St. George's for a tumour in the right breast. Seven years previously she was operated on by Sir Astley Cooper, at Guy's, for a scirrhous tumour in the same breast. The present tumour commenced soon after the operation abovementioned, and was now the size of an egg, hard, irregular, scirrhus-like, free and moveable, with only an attachment to the integuments above. The general health appeared good, and the uterine functions had ceased. Some of the absorbent glands in the axilla were slightly enlarged, but not at all indurated. At her own particular request, the tumour was removed, and the glands in the axilla subsided to nearly their natural size. She was discharged cured in less than two months. One year afterwards the patient returned, "in the last stage of jaundice," being much emaciated, and complaining of occasional acute pain in the epigastric and hypogastric regions. The liver could be felt enlarged. She died in a month from this period.

*Dissection.* Several small, white, hard tubercles were found under the integuments of the thorax, and a few in the pleura costalis. The lungs were sound. Some serum was effused in the abdomen. The liver was enlarged, indurated, and full of large white tubercles. Some of the mesenteric glands were enlarged and hardened, and a few tubercles were observed under the peritoneal covering of the intestines and bladder.

It is a pity that Mr. Jeffreys did not state what was the condition of the gall-bladder and ducts; and whether the jaundice was occasioned by mechanical obstruction to the course of the bile, or by the organic disease of the liver, as this is a litigated point of pathology at the present moment.

*Case* 2. This was an aged female of 70, who came into the Hospital, in May, 1826, with a small scirrhous tumour in the left mamma—an indurated mass of glands in the axilla—œdema of the arm—prominence and pain of the abdomen—pain over the sacrum—irritation in making water—furred tongue, and quick pulse. She was, of course, no subject for a surgical operation, and remedial treatment had no other effect than, perhaps, prolonging a wretched existence for six or seven months, when she died.

*Dissection.* The tubercles in the breast and axilla presented the genuine characters of scirrhus. There was a large tumour in the abdomen exhibiting the same kind of morbid structure, and involving many parts in a mass of adhesive inflammation. Between the rectum and sacrum was another tumour, the size of an orange, embracing the posterior half of the gut, and contracting its calibre. There were also some tubercles in the liver.

This woman only dated her complaints some eight months before she applied for relief at the hospital.

*Case* 3. A female, aged 44, had had a tumour in the mamma for two years. It presented the character of scirrhus, and some of the axillary glands were enlarged and indurated. She complained of darting pain through the breast, and occasional pain and numbness of the arm. There were two small ulcerations on the mamma. In a week after admission, she was seized with severe erysipelas of the diseased breast, which spread to the side and back, of which she died on the third day.

*Dissection.* The mammary tumour was genuine scirrhus, and those in the axilla were white tubercles. Within the thorax, immediately under the diseased breast, there was a scirrhous tumour, as large as half an orange, situated between the ribs and pleura.

Although these and many other cases which might be cited show the general disposition to scirrhous affections in some constitutions; yet, while the ablation of a scirrhous tumour is even occasionally successful, that *ultimum remedium* must be applied, in the absence of palpable proof that this disposition exists.

---

### 15. EXTIRPATION OF THE PAROTID GLAND.

[M. Lisfranc. La Pitié.]

We no longer hear it asserted by eminent surgeons, that the parotid gland cannot be entirely extirpated. We have placed on record, in this Journal, several well-attested cases, and are now about to add another to the list. The operation was performed by M. Lisfranc, the distinguished surgeon of La Pitié.

*Case.* J. Prevost, aged 60 years, was received into hospital, on the 1st of June, 1826, on account of a fibrous tumour, the size of a man's fist, occupying the region of the parotid gland, and extending beyond that in several directions. Although the tumour was moveable in some directions, it was fixed in others, shewing that its roots penetrated deep, and were adherent to the neighbouring bones. After a few days of proper regimen, the operation was performed, in La Pitié, on the 5th June. We need not detail the steps of the operation with any degree of minuteness—since the same process will rarely be applicable to two cases. After elliptical incisions had been made, so as to embrace the tumour, and some dissection had been gone into around it, during which several vessels were divided, it was attempted to insulate the disease by the fingers and by the handle of the scalpel; but this was found to be quite impracticable, on account of the number and strength of the ligamentous attachments to the surrounding parts. The surgeon was therefore obliged to have recourse to the knife. A tedious, painful, difficult, and dangerous dissection ensued, but was ultimately crowned with complete success, in the removal of the whole of the diseased parts, including the parotid gland. Some of the attachments went to the transverse processes of the first two cervical vertebræ, and to the stylo-mastoid process. The carotid artery was completely insulated for some space, and no sign of pulsation could be seen or felt, unless the artery was compressed between the fingers, when the rush of blood through the vessel, at each systole of the heart, communicated the sensation to which the term pulse has been applied. One attachment of the tumour was to the condyle of the lower jaw, and others insinuated themselves between the pterygoid muscles. These were all carefully separated; and in this part of the dissection, a large and powerful column of blood sprang to the height of several feet from the bottom of the wound! It was overcome by pressure. The operation was continued amidst appalling hæmorrhage, by which the patient was, for a time, deprived of sense. After he had revived a little, M. Lisfranc kept pressure on the internal maxillary artery, which was divided, while he proceeded to tie, seriatim, the temporal, transverse facial, auricular, and mastoid arteries. The internal maxillary (ou la terminaison de la carotide externe) remained to be secured. With equal difficulty and dexterity, this intrepid surgeon seized and tied this great vessel, amidst deluges of blood; and the operation was finished. The wound was dressed, and the patient put to bed. We need not follow the diurnal reports of the case afterwards. The patient went on remarkably well till the 27th of the same month, the wound gradually filling up. At this time, whether from some dietetic error or other cause, erysipelas of the face took place, attended with vomiting and diarrhœa. Amongst matters thrown up were some preserved cherries. Fever now became developed, and there were evident symptoms of abdominal inflammation. This last was conquered by numerous leeches—the suppuration went on kindly—and the wound was healing fast. On the 2d July a relapse took place, and some blood appeared in the stools. After various vacillations, during which the erysipelas appeared and disappeared several times, a colliquative diarrhœa came on, and could not be suppressed. He was carried off by this complaint on the 16th July, six weeks after the operation.

On dissection, the mucous membrane was found inflamed throughout its whole extent, and several large ulcerations were discovered, some of which had penetrated through the muscular and peritoneal coats, and caused extravasation of the intestinal matters into the general cavity of the abdomen. This, of course, was the cause of death. The preparation of the extirpated tumour, including the whole of the parotid gland, was presented to the Academy of Surgery, and there examined by numerous members, who verified the statement of the extirpation. The examination of the part where the operation had been performed, corroborated also the truth of the details already given. It is evident that, as far as the operation was concerned, the surgeon was successful. If he could not control the gastro-enteritic inflammation and ulceration that supervened, there was no blame attached to the surgery of the case. —Revue Medicale, December, 1826.

### 16. INDURATION OF THE CELLULAR TISSUE.

[South London Dispensary.]

We have, on several occasions, given some accounts of this curious disease, chiefly from foreign authors. Dr. Mac Andrew has recently published an instance of this complaint as it occurred in his dispensary practice. The patient was a boy, 18 months old, who had become feverish about two months previously, and then affected with a diarrhœa and swelling of the lower extremities. These last complaints never left him. The feet, legs, and thighs present a diffused swelling, rendering the skin tense. This swelling has a wax-like appearance, and is nearly colourless, except at one spot, where it has a livid tint. It does not pit, nor is there any pain, on pressure. All the affected parts are cold to the touch. The body is emaciated—the bowels moved nearly twelve times a day. The motions are of yellow, sometimes greenish hue. Some cretaceous powders were given, and a lotion of spirit and mindererus applied to the parts. In two days the swelling had greatly diminished. He died, however, in a couple of days afterwards. There was nothing unusual in the chest. In the mucous membrane of the bowels there were several portions bearing marks of phlogosis. The cellular membrane of the left leg was much thickened, of a light red colour, dense and unyielding on pressure, presenting a granular appearance, not unlike hepatised lung. Below this there is a layer of gellatinous substance, two lines in thickness, containing a fluid. The muscles were unaffected.

The causes of this strange affection are unknown. The French attribute it to cold. It is generally fatal, and the treatment uncertain. Dr. Underwood almost always found the complaint connected with diarrhœa. The improvement of the general health seems to be the main indication. Friction, pressure, and gentle mercurials may assist in removing the cellular induration.—*Med. and Phys. Journ. No.* 8, *N. S.*

### 17. DEUTO-IODURE OF MERCURY.

[St. Louis.]

The union of iodine with mercury is now obtaining a trial at the St. Louis Hospital, and other establishments, in various cutaneous affections, and is evidently a most powerful application, which bids fair to be of considerable service in certain diseases of the skin, which resist other means, and so often foil all the efforts of the practitioner. Dr. Paillard has lately read a memoir on this subject, at the Royal Academy of Medicine, of which we shall here give some account.

This medicine is prepared by mixing a solution of hydriodate of potash with a solution of deuto-nitrate of quicksilver, when a copious red precipitate falls to the bottom. The nitric acid unites with the potash, while the iodine combines with the mercury, and forms deuto-iodure of quicksilver. It is soluble in a certain proportion of sulphuric, nitric, and acetic ether. One ounce of sulphuric ether will dissolve 14 grains of the deuto-iodure. By evaporation we obtain beautiful red crystals, of a parallelogramic form. The other ethers do not dissolve so large a proportion. This medicine may be used internally and externally; but it is with the external application that we have now to do. It may be employed either dissolved, as above, or mixed with hog's lard, in the way of an ointment. The ointment used by M. Biett, consists of 15 grains of deuto-iodure of mercury—two ounces of lard, and 20 drops of essence of bergamotte.

Our author first used the deuto-iodure dissolved in ether, applying it by means of a hair-pencil passed twice or thrice over the parts. The effects were as follows: first, a sensation of cold is perceived, by the evaporation of the ether—next is formed a kind of red crust, by the precipitation of the iodure, causing sometimes a smarting pain, and, in a few instances, small blisters. Ultimately there is a desquamation of the cuticle, with some clefts or indentations. If the brush is passed a good number of times over a part, pain, swelling, and vesication succeed, in the course of a few hours, ultimately followed by a kind of scab, like the crusta lactea of children. These crusts fall off in two or three days, and then the dartrous or other cutaneous affection for which the deuto-iodure was applied, is found to be beneficially modified, and in a state more favourable for the process of cure. Used in this manner, the medicine gives very little pain, and never produces any constitutional disturbance. By such applications our author has seen various cutaneous eruptions removed, and old and rebellious ulcerations healed. But, for many purposes, this form is too mild, especially in cutaneous diseases of long standing, and in engorgements of the glands and cellular membrane of any magnitude. On this account, our author mixed a drachm of the deuto-iodure in an ounce of oil of almonds, expecting by this method to have a preparation possessing considerable caustic powers. At first he applied this liniment with caution, and over a small surface, but afterwards he grew bolder. The layer applied should be in proportion to the degree of cauterisation which is wished. At the first moment of application, the patient feels no pain or heat; but gradually the action of the caustic begins to be perceived. In eight or ten minutes a sense of heat is felt, which increases and changes into burning pain. The neighbouring parts swell, and become hot and painful. In the course of an hour, there is often a discharge of limpid serum from the part. The pain abates in proportion as the application is absorbed or becomes dry. It generally ceases entirely in the course of four or five hours, leaving only some swelling, which is usually dispersed in the course of one, or, at the most, two days. In a very few cases, the pain of the caustic lasted six or seven hours, and the subsequent swelling two or three days.

In some cases, where the application was made on the face, there came on a sudden salivation, an hour or two after the application was made. M. Manry saw two instances of this kind, in his own practice. The patient may, at any time, mitigate the pain of the caustic, by exposing the part to a current of air, or by fomenting it with any emollient fluid. These fomentations may be made without any danger of washing off the preparation, as this form of the caustic adheres tenaciously to the parts.

This preparation is partly absorbed, and partly desiccated. It combines with the most superficial tissues, producing crusts or scales, which fall off in three or four days, either spontaneously, or by the aid of a fomentation or poultice. The surface beneath generally presents a vermillion aspect, and is strongly disposed to cicatrisation. Our author has seen instances where, in two or three days, the skin being covered with dartrous ulcerations, the cellular substance gorged, thickened, and studded with enlarged glands—the swelling disappeared, the engorgement greatly diminished, and the pain and irritation previously existing ceased.

In scrofulous swellings the caustic effects of this application are little perceptible, during the first or second trial; but absorption appears to commence before any cauterisation obtains. In the course of the third or fourth application to indolent scrofulous swellings, pain and inflammation are produced, along with the other phenomena described above, and then the absorption and diminution of the original engorgement become daily perceptible.

It is to be observed, however, that, in some instances, certain constitutional symptoms were excited, such as fever, malaise, colicky pains in the bowels, and diarrhœa. In no instance did these prove serious; but still they shew that the medicine is a powerful one, and that part of it is absorbed. Our readers will readily see that this application may be used for several purposes, where we now apply common caustic and the potential cautery. We have not space for the cases treated at the St. Louis by our author. They are published in detail, in the Nouvelle Bibliotheque, for December, 1826.

---

### 18. AMAUROSIS CURED BY LONG-CONTINUED VOMITING.

[Hospital of Florence.]

This curious case is related by Professor Polidoro, of Florence, and the particulars are as follow:—A female, aged 30 years, of pale lymphatic temperament, had experienced, when only seven years old, a long febrile affection, together with convulsions, and paralysis of some of the members. When these phenomena disappeared, there came on a severe pain in the right orbit and neighbouring parts, with complete blindness of that side, nothing appearing wrong in the coats or humours of the organ. This pain continued till the patient was 20 years of age, when she happened to fall down a flight of stairs. This accident was followed by an abundant discharge of muco-purulent matters from the nostrils, and then the pain in the orbit ceased. In the beginning of Nov. 1825, she began to experience pains, sometimes in one, sometimes in the other side of the head, accompanied by a sense of tension. Towards the end of the same month, the *left* eye became the seat of a very severe pulsating pain, soon followed by blindness of that eye also, so that she could now scarcely discern light from darkness. Blood-letting, local and general, was several times practised, together with blisters, &c. but no relief had been obtained. On the 9th December, Professor Polidoro examined the patient attentively, and nothing particular could be observed in the state of the eyes, except dilatation of the pupils, with insensibility to the brightest light, blindness, squinting, and a pale corneous spot at the bottom of the right eye. She refused to have a seton, but took a grain of tartar-emetic in eight ounces of the infusum arnicæ montanæ. 11*th*. Two grains of the tartrite were given in the same vehicle —vomiting induced. 12*th*. The same medicine, and the same effect. Some vision in the *left* eye. 13*th*. The same medicine. After vomiting, she experienced a tensive pain at the bottom of the orbits. Liquid stools followed the vomiting. 14*th*. Same treatment. She could now see to thread a needle with the left eye. The same medicine was continued. 17*th*. She begins to have some slight vision with the *right* eye, which had been blind for more than 20 years. The nitrate is continued, but does not produce vomiting. 19*th*. The dose is increased to three grains of the tartrite daily. 20*th*. Several vomitings by this last dose. She can distinguish some objects faintly by means of the right eye alone. 24*th*. She could now read with the *left* eye, and could distinguish letters of a certain size with the *right*, but not in their proper place. 25*th*. The pupils contracted to their natural size, and were obedient to light. The patient less frequently vomited than before. 26*th*. She could see the buttons on a person's coat with the right eye, and the sight of the left was nearly as good as ever. She now demanded permission to go from the hospital, and she was discharged towards the end of February, 1826. She has since been seen, and continues to preserve her sight.—*Journ. Complem. Nov.* 1826.

It is evident that the optic nerves and retina, in this case, were not disorganized, but only their functions prevented by some deposition which has been removed by the long-continued vomiting which was perseveringly employed, and most patiently submitted to. The powerful absorption which obtains under the action of emetics, is not sufficiently appreciated in this country—or, at least, we do not avail ourselves of this mode of exciting the absorbents so much as we ought to do. When we speak of *exciting the absorbents*, we know that we are treading on tender ground; but we are speaking practically rather than physiologically; and, on such terms, we say that vomiting *increases absorption*—whether by directly or indirectly *exciting* the absorbents, is not very material.

